# An arch-bridge-type fluorophore for bridging the gap between aggregation-caused quenching (ACQ) and aggregation-induced emission (AIE)†

**DOI:** 10.1039/c6sc01254j

**Published:** 2016-03-29

**Authors:** Manna Huang, Ruina Yu, Ke Xu, Shuxian Ye, Shi Kuang, Xinhai Zhu, Yiqian Wan

**Affiliations:** a School of Chemistry and Chemical Engineering, Sun Yat-sen University Guangzhou 510275 P. R. China ceswyq@mail.sysu.edu.cn

## Abstract

Solution and solid dual photoluminescence (PL) molecules fill the substantial gap between ACQ and AIE molecules to explore the mechanism of molecular luminescence in greater detail and to facilitate practical applications. A unique arch-bridge-like thiazolo[5,4-*b*]thieno[3,2-*e*]pyridine moiety is obtained as a stator after the rigidification of rotor 1 by intramolecular H-bonding of *ortho* –OH or –NH_2_ to afford two classes of solid and solution dual PL molecules. As a typical example, DF5 is dual PL active. Moreover, the large Stokes shift with high dual PL efficiency (*Φ*_F_ up to 51% in the solid state, 80% in DMF, 74% in DMSO, and 100% in water), together with the good thermal stability (*T*_m_ > 200 °C and *T*_05_ > 200 °C), make it more practical for promising optoelectronic and biological applications.

## Abstract 2

A novel unique arch-bridge-like stator, after the rigidification of rotor 1 by intramolecular H-bonding, afforded two classes of solution and solid dual photoluminescence (PL) molecules.

## Graphical abstract

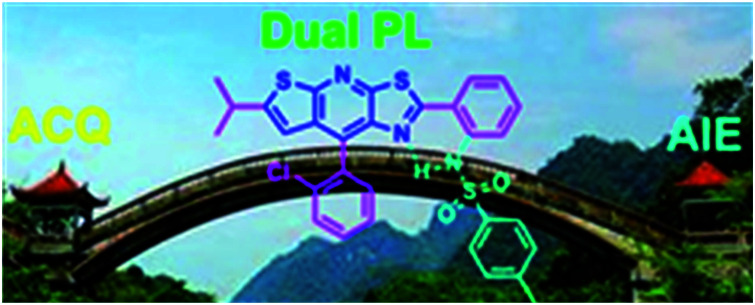

## Introduction

The design and synthesis of efficient organic luminescent molecules are currently of interest in industry and academia because of their potential applications in electronics, photonics, optoelectronics, chemosensors and bio-probes.^1,2^ Traditional organic fluorophores that consist of planar and polycyclic π-conjugated frameworks generally exhibit high-efficiency luminescence in dilute solution. Nevertheless, severe luminescence quenching of the fluorophores usually occurs in highly concentrated solutions or in the solid state because of non-radiative pathways in the short-range molecular interactions, such as π–π stacking originating from the planarity of the molecular skeleton. This phenomenon, known as aggregation-caused quenching (ACQ),^3,4^ was first observed in the fluorescence of pyrene by Forster and Kasper,^5,6^ and often hampers such practical applications as optoelectronic devices, imaging agents and biosensors.^7^

To circumvent these limitations, numerous approaches, including molecular planarization, restriction of intramolecular rotation (RIR), prevention of exciton diffusion, efficient energy transfer from monomers to aggregates, J-aggregate formation, and synergistic combinations of these effects, have been employed to prevent or alleviate luminophore aggregation.^8,9^ However, these efforts have had only limited success because of the intrinsic aggregating nature of planar polycyclic luminophores located close to each other in the condensed phase, until Tang and his co-workers reported their pioneering work in 2001.^10^ Briefly, 1-methyl-1,2,3,4,5-pentaphenylsilole (MPS) was found to be luminescence active after solvent evaporation on a thin-layer chromatographic plate under UV light, and thus, the concept of aggregation-induced emission (AIE) was introduced. Since then, a large variety of AIE or AIEE (aggregation-induced emission enhancement) molecules with twisted shapes have been obtained,^11–33^ and the RIR mechanism has been demonstrated to be the main cause of the AIE phenomenon.^10,34–43^ The discovery of the AIE and AIEE effect overturned the general beliefs regarding the ACQ of luminescence processes and opened up a new avenue for the development of novel luminogenic materials for diverse applications in the aggregate or solid state.^2,11,13–16,33,44–61^

It must be noted that AIEE molecules can emit both in solution and in the aggregated state, however, their fluorescence intensity or fluorescence quantum yield in the solid state is usually much higher than that in dilute solution.^62^ Hence, there has still been a huge interest in the development of highly luminescent materials, both in solution and in the solid state, so as to explore the mechanism of molecular luminescence in more detail and to facilitate the practical applications, especially in complicated bioassay systems.^63,64^

In fact, some pioneering works on solution and solid state dual photoluminescence (PL) have recently been reported.^62–75^ For example, Tang and his co-workers coined the concept of the conjugation-induced rigidity (CIR) strategy for the design of molecules with dual photoluminescence.^63^ While Xu *et al.* established smart lanthanide bio-probes by equipment of both antennae with the AIE and ACQ effects, which can be used in complicated bioassay systems.^64^ However, the solution and solid dual photoluminescence molecules, in particular those containing novel fluorophores, even including AIEE molecules are still limited to several classes in comparison with AIE and ACQ molecules.^76^ Herein, we report our work on the design and synthesis of a novel type of solution and solid dual luminogen molecules.

## Results and discussion

**Fig. 1:**
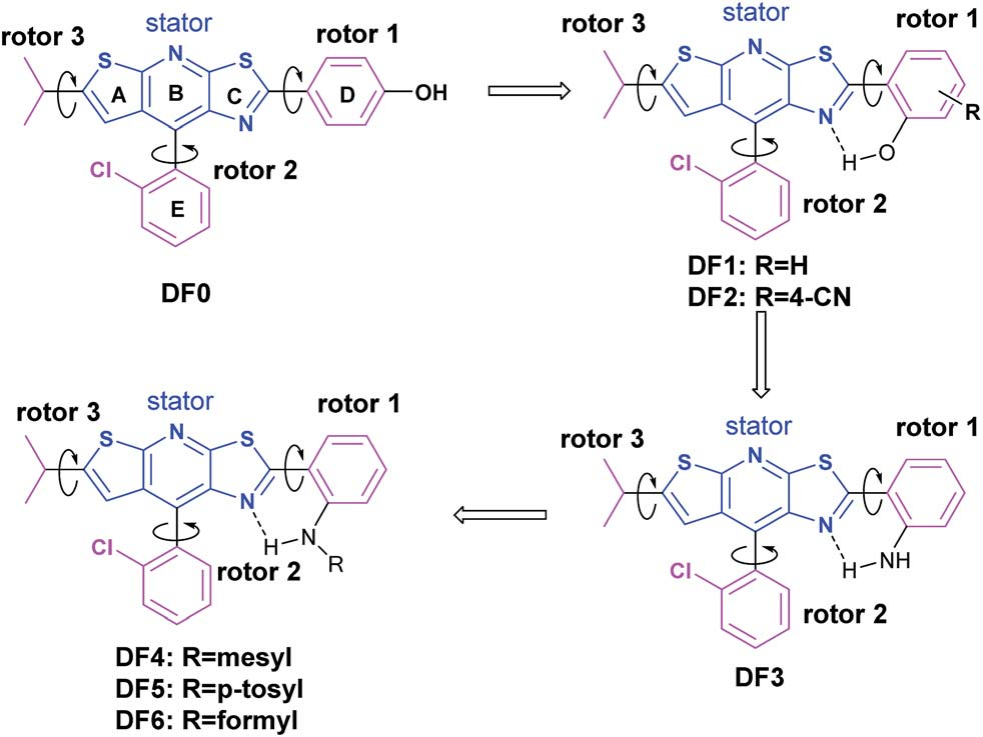
Design of the compounds.

In general, intramolecular rotation and conjugation are two key parameters in the design of AIE and ACQ molecules, respectively. Hence, we assumed that the balance between the rotation and conjugation effects was important for the development of the solution and solid state dual luminescence molecules. Moreover, we recently found that unique thiazolo[5,4-*b*]thieno-[3,2-*e*]pyridine derivatives can be readily obtained in a one-pot synthesis.^77^ These derivatives can be roughly regarded as isosterically substituted acridines with interesting non-complete planar fused triheterocycle (rings A–B–C) geometries. This unique arch-bridge-type geometry attracted our attention for the development of solution and solid dual photoluminescence (PL) molecules because their non-complete planar geometry should resist the π–π stacking effect (Fig. 1, DF0).

**Scheme 1:**
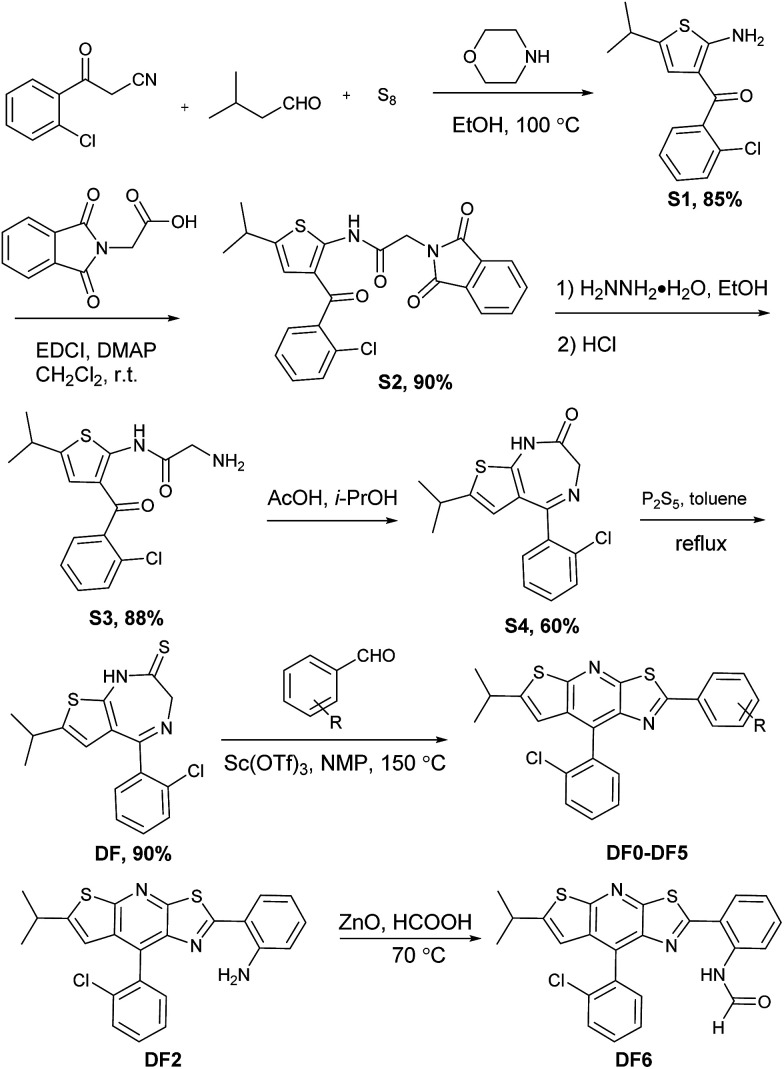
Synthesis of target compounds.

The synthesis of the thiazolo[5,4-*b*]thieno[3,2-*e*]pyridine derivatives (DF0–DF6) is outlined in Scheme 1. Compound DF, a key intermediate, was synthesized from commercially available starting materials: 3-(2-chlorophenyl)-3-oxopropanenitrile, powder sulfur and isovaleraldehyde, according to a previously reported protocol.^77^ The target compounds DF0–DF5 were synthesized by reacting DF with the corresponding aldehydes, with Sc(OTf)_3_ as the catalyst in *N*-methyl-2-pyrrolidone (NMP). The target compound DF6 was synthesized by reacting DF2 with ZnO as the catalyst in formic acid.

DF0 initially showed weak PL efficiency in dimethylformamide (DMF), benzene and tetrahydrofuran (THF), with values of *Φ*_F_ > 10% and very weak solid PL efficiency (*Φ*_F_ only 1%) (Table 1, DF0). The non-radiative energy-dissipating paths of the excitons of DF0 resulted from intermolecular hydrogen bonding and photo-induced proton transfer that decreased the PL efficiency in solution, whereas intramolecular rotations (3 rotors) and intermolecular π–π interactions caused the weak solid PL efficiency. X-ray diffraction analysis of single crystals from EtOH confirmed that two DF0 molecules were bridged to a dimer through an EtOH by two intermolecular hydrogen bonds (Fig. S1†). The intramolecular hydrogen bonds of luminogens have been recognized to rigidify the molecular structure and are extensively utilized to activate the RIR process for the development of AIE molecules.^9,78,79^ Hence, we moved the *para* –OH to the *ortho* position of ring D in DF0 to construct a new molecule (DF1), in which the intramolecular H-bonding restricts rings C and D. As was expected, DF1 exhibited moderate PL effects in both solution (dimethyl sulfoxide (DMSO) and DMF) and the solid state, with *Φ*_F_ values exceeding 20% (Table 1, DF1). The decreased torsion angle (from 19.1° in DF0 to 6.7° in DF1) between rings C and D indicated stronger rigidity and led to greater PL efficiency in solution. Moreover, the intramolecular H-bonding restricted the rotation of rotor 1 and raised solid PL efficiency, although the intermolecular π–π and CH⋯π interactions resulting from head-to-tail J-aggregation (*i.e.*, the distances between the ring's centroids were less than 4 Å) hindered the solid PL to a certain extent (Fig. S2†).

**Table 1:** Optical properties of the compounds

	Solvents	*λ*_abs_ [nm]	*λ*_em_ [nm]	*Φ*_F_ [%]	*τ* [ns]
DF0	Benzene	360, 344, 289	387	10	0.28
	THF	361, 346, 290	392	12	0.18
	DMF	363, 348, 292	405	15	0.22
	DMSO	445, 363, 350, 293	588	0	3.73
	Solid	394	452	1	0.46
DF1	Benzene	373, 356, 318, 283	545	2	0.23
	THF	370, 354, 280	395	1	0.42
			545	3	0.10
	DMF	460, 366, 352, 281,	568	20	4.71
	DMSO	458, 366, 350, 282, 258	568	24	4.32
	Solid	374	550	26	4.11
DF2	Benzene	385, 365, 329, 288	540	41	3.51
	THF	380, 364, 329, 288	540	8	1.15
	DMF	470, 327, 272	495	77	3.93
	DMSO	470, 328, 263	495	90	3.82
	Solid	440	564	40	3.95
DF3	Benzene	390, 321, 284	455	13	0.70
	THF	398, 319, 283, 269	465	72	1.33
	DMF	405, 273	483	49	2.94
	DMSO	405, 320, 284	490	48	3.07
	Solid	400, 283	525	2	0.94
DF4	Benzene	356, 323, 285	583	0	0.43
	THF	355, 321	586	3	0.11
	DMF	425, 352, 317, 266	544	98	5.43
	DMSO	425, 356, 322, 283	547	72	5.95
	Solid	390	572	39	5.49
DF5	Benzene	356, 322, 284	578	7	0.68
	THF	356, 322, 284	580	6	0.05
	DMF	420, 352, 312, 266	535	80	5.48
	DMSO	420, 352, 313, 259	538	74	5.59
	Dioxane	355, 321, 283	575	34	0.44
	MeOH	352, 320, 282	570	17	0.17
	H_2_O (pH = 7.4)	359, 325, 283	570	100	7.09
	H_2_O (pH = 1.9)	382, 363, 292	570	100	5.83
	H_2_O (pH = 12.8)	361, 281, 253	565	25	3.88
	Solid	410	571	51	5.29
	Glasses	355	570	14	4.66
DF6	Benzene	375, 358, 325, 286	410	2	0.83
			582	13	0.80
	THF	372, 356, 324, 285, 255	410	3	0.22
			582	6	0.12
	DMF	371, 356, 324, 285, 263	426	0	0.37
			580	7	0.10
	DMSO	356, 325, 285, 255	426	2	0.31
			581	3	0.26
	Solid	395	475	11	1.70

To explore the effect of elongation of the conjugate structure on the PL efficiency, the cyano-containing compound DF2 was synthesized. The introduction of a cyano group usually resulted in weaker PL efficiency in solution because of the steric effect and intrinsic intramolecular charge-transfer (ICT) process.^63^ However, DF2 exhibited much stronger PL efficiencies in solution (*Φ*_F_: DMF, 77%; DMSO, 90%; and benzene, 41%) and the solid state (*Φ*_F_: 40%) (Table 1, DF2). These results suggested that the elongation and greater rigidity of the conjugation system, which was confirmed by the decreased dihedral angles of the arch-bridge rings (A–B and B–C), led to high PL efficiency in solution; whilst the weak intermolecular π–π interaction (rings C–C′ and B–C′) between monomers resulted in a good solid PL efficiency (Fig. S3†).

To explore the effect of intramolecular H-bonding on the PL efficiency, we designed and synthesized DF3 by replacing the H-bond donor –OH with –NH_2_. For steric reasons, the –NH_2_ group was twisted out of the plane of the fused aromatic ring.^9^DF3 was a classical ACQ molecule, with only 2% *Φ*_F_ in the solid state and 72% *Φ*_F_ in THF because of weak intramolecular H-bonds that were unable to confine, effectively, the rotation of rotor 1 and rotor 2. Solid aggregation was not studied further because this compound is too labile to afford useful single crystals. As a general rule, highly twisted conformations in the solid state are unable to achieve the close intermolecular contact needed to offer high solid PL efficiency. Another hydrogen atom of the –NH_2_ was substituted with a bulky group to generate another arm and enhance the possibility of J-type aggregation and further molecular self-assembly by partially avoiding the overlap (H-aggregate) between neighbouring dimers.^9^ Hence, DF4, a methylsulfonamide, was synthesized and found to be a good basis for further work: DF4 displayed good solid PL efficiency (*Φ*_F_: 39%) and excellent PL efficiency in DMF (*Φ*_F_: 98%) (Table 1, DF4). The decreased PL efficiency in DMSO (*Φ*_F_: 72%) compared with that in DMF was intriguing. This phenomenon resulted from the stronger H-bond-donating capability of DMSO, as confirmed by observing a pronounced intensity difference of the absorption spectrum in DMF and in DMSO (the first maximum peak, *λ* = 425 nm, corresponding to the H-bond-donating effect of the solvent) (Table 1, DF4; and Fig. S7,†DF4). This compound's good solid PL efficiency originated from the crystal packing style of DF4, namely, two DF4 molecules assembled in a cube cavity-type dimer, in which the intramolecular rotation of rotor 1 was restricted by the rigid structure. However, only a partial J-aggregation or a distorted J-aggregation (3.93 Å between C and C′ centroids and 3.69 Å between C and D′, Fig. S4†) persisted because of the red-shift of the absorption band of only 35 nm (from in THF to the solid state, Table 1, DF4; and Fig. S7,†DF4).^80^

**Fig. 2:**
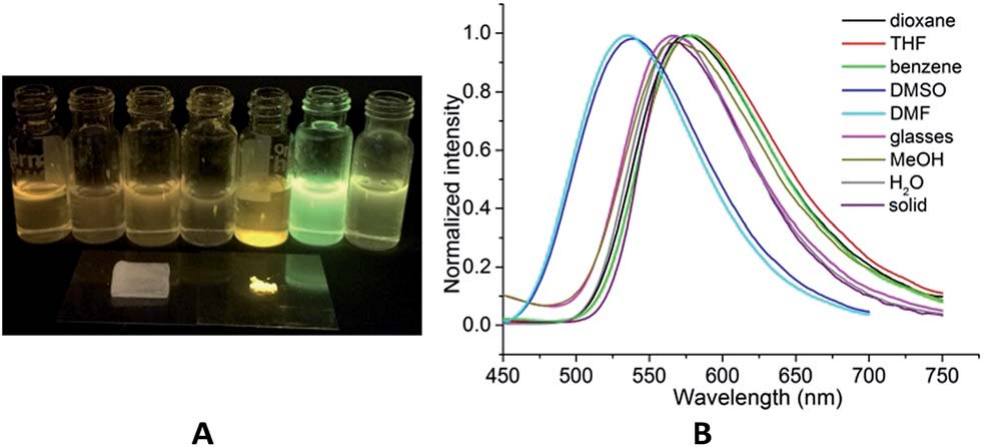
(A) Photographs in solution (2 × 10^−5^ M, from left to right: benzene, THF, dioxane, MeOH, H_2_O, DMF and DMSO), glasses and crystal states of DF5 taken under 365 nm UV illumination. (B) Normalized emission spectra of DF5.

**Fig. 3:**
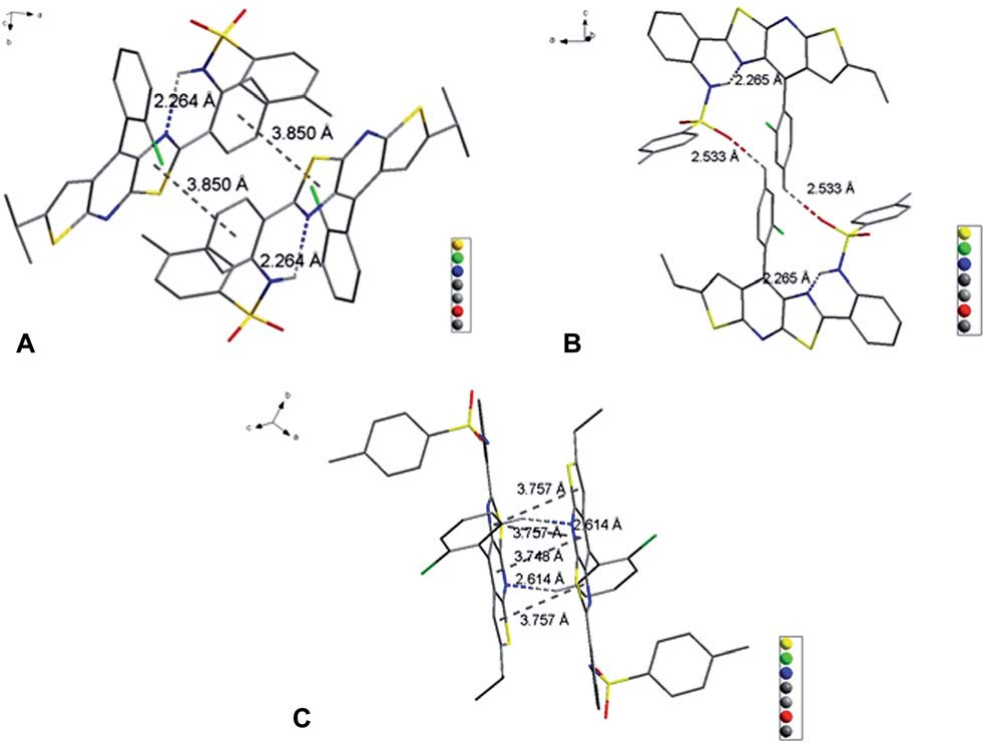
Molecular interactions of DF5 in single crystals.

Slightly modifying DF4 by replacement of methylsulfonyl with 4-methylbenzenesulfonyl to afford DF5, generated greater solution and solid PL efficiencies (Fig. 2). The significant increase in the *Φ*_F_ values from 39% (DF4) to 51% (DF5) in the solid state is explained by the distorted J-type aggregation packing mode resulting from arch-bridge-like π-conjugation fused tricycles. As shown in Fig. 3, the vertical distance is approximately 3.8 Å between cavities and between the sides inside the cavities. The intercavity π–π interaction formed by the anti-parallel monomers disrupted the solid PL efficiency, whereas the intracavity π–π interaction was decreased by the staggered floor packing mode, increasing the PL efficiency. However, the intramolecular rotation of rotor 2 was restricted because of the weak H-bonding between the oxygen atom of the sulfonyl group and the C–H of *o*-chlorophenyl together with the steric hindrance of the bulky 4-methylbenzenesulfonyl group. As a result, the unique rigid cavity-shape assembly blocked the non-radiative pathway in the solid state, leading to high solid efficiency.

To distinguish the RIR effect from the possible aggregation-induced packing effect as the primary cause of the solid PL efficiency, DF5 was dispersed in rigid, room-temperature sucrose octaacetate (SOA) glasses at concentrations of approximately 0.01 mM, and its PL efficiency was determined.^81^ The relatively low *Φ*_F_ value (14%) indicated that the main cause of the high solid PL efficiency of DF5 should be from some special mechanism, for example the J-aggregation effect, instead of the usual molecular RIR mechanism, which was confirmed by the obvious red-shifted absorption band (from 355 nm in THF to 410 nm in the solid state, Fig. S7,†DF5).^80^ Moreover, the greater rigidity of the conjugated tricycle (confirmed by the decreased dihedral angles: A–B, 0.96° and B–C 1.76°) made the PL active in various solutions (*Φ*_F_: DMF, 80%; DMSO, 74%; dioxane, 34%; THF, 6%; MeOH, 17%; H_2_O, 100%; and benzene, 7%) (Table 1, DF5). In addition, evidence for specific solvent–fluorophore interactions can be seen in aprotic solvents such as DMF and DMSO from the occurrence of new bands (*λ*_max_ = 420 nm) in the absorption spectra and the obvious blue shift (*ca.* 40 nm) in the emission spectra. This suggested that the specific solvent–fluorophore interactions occurred in either the ground state or the excited state due to the H-bond donor capacity of aprotic polar solvents. In contrast, there were no obvious changes in both absorption and emission spectra in protic solvents, such as MeOH and water, compared to that in benzene. Furthermore, proton transfer had little effect on the emission spectrum.

**Fig. 4:**
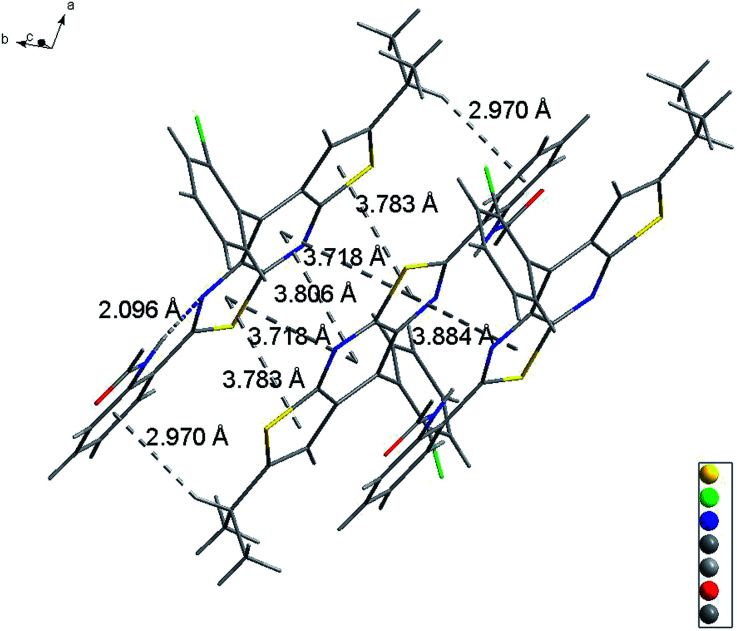
Molecular interactions of DF6 in single crystals.

To explore further the mechanism of the high solid PL efficiency of DF5, we designed and synthesized DF6 by supplanting the bulky 4-methylbenzenesulfonyl with a formyl group to alleviate the steric hindrance. As was shown in Fig. 4, the unique rigid cavity-shaped assembly was not retained and the rotation of rotor 2 would be easier without the H-bond between the oxygen atom of the formyl group and the C–H of *o*-chlorophenyl, although corresponding intermolecular interactions are similar to those in DF5 and DF4. As a result, poor solution and solid PL efficiencies of DF6 were observed (Table 1, DF6).

The large Stokes shift (largely exceeding 100 nm) of DF5 with relatively high PL efficiency in the solid state and in some solutions resulted from the intramolecular hydrogen bonding effect and the polarity effect, which facilitates the detection of fluorescent species when used as a fluorescent probe.^82,83^

Finally, the thermal properties were investigated by differential scanning calorimetry (DSC) and thermogravimetric analyses (TGA). All *T*_m_ values and *T*_05_ values were found to exceed 200 °C (Fig. S12, Table S2†). The relatively high thermal stability suggested that this species has potential practical applications as a luminescent emitter because thermal stability governs the stability and lifetime of such devices.

## Conclusions

In conclusion, we discovered a novel and unique fluorophore with an arch-bridge-like thiazolo[5,4-*b*]thieno[3,2-*e*]pyridine structure as a stator after the rigidification of rotor 1 by intramolecular H-bonding of the *ortho* –OH or –NH_2_ to afford two classes of solid and solution dual fluorescent molecules. The typical example, DF5, was solution and solid dual PL active with maximum emissive wavelengths at approximately 571 (solid), 535 (DMF), 538 (DMSO), 578 (benzene), 580 (THF), 570 (MeOH), 570 (H_2_O), and 575 (dioxane) nm. Moreover, the unique cavity geometry resulting from the intermolecular assembly was the main cause of the good solid PL efficiency of DF5. Additionally, the large Stokes shift with high dual PL efficiency (*Φ*_F_ up to 51% in the solid state, 80% in DMF, 74% in DMSO, and 100% in water), together with good thermal stability (*T*_m_ > 200 °C and *T*_05_ > 200 °C), made this molecule relatively practical for optoelectronic and biological applications. More effective dual PL molecules will be designed and synthesized in our laboratory based on the concept: (1) rigidifying arch-bridge-like type stators with other rotors (*e.g.*, rotor 3) by various approaches, including intramolecular H-bonding or bulk substituents, to obtain high solution PL efficiency and (2) controlling intermolecular assembly to restrict RIR and avoid H-aggregation to achieve high solid PL efficiency.

## Supplementary Material

SC-007-C6SC01254J-s001

SC-007-C6SC01254J-s002
