# Supplementary material for: An arch-bridge-type fluorophore for bridging the gap between aggregation-caused quenching (ACQ) and aggregation-induced emission (AIE)
Source: Chem Sci. 2016 Mar 29;7(7):4485–91. doi: 10.1039/c6sc01254j (PMC6016330; doi:10.1039/c6sc01254j)
Supplement: SC-007-C6SC01254J-s001 [file SC-007-C6SC01254J-s001.pdf]

Electronic Supplementary Material (ESI) for Chemical Science.
This journal is © The Royal Society of Chemistry 2016

An arch-bridge-type Fluorophore for Bridging the Gap Between Aggregation-caused Quenching and Aggregation-induced Emission

Manna Huang, Ruina Yu, Ke Xu, Shuxian Ye, Shi Kuang, Xinhai Zhu* and Yiqian Wan*

School of Chemistry and Chemical Engineering, Sun Yat-sen University, Guangzhou, P. R. China, Fax: 8620 8411 3610, E-mail: ceswyq@mail.sysu.edu.cn

Contents

1. **General methods** 2
2. **Synthesis of the compound DF** 2
3. **General Procedure for syntheses of target compounds** 5
4. **Preparation of room temperature glasses** 8
5. **Properties of the single crystal structures** 9
6. **UV absorption spectra of the target compounds** 14
7. **Excitation spectra of the target compounds** 17
8. **Emission spectra of the target compounds** 19
9. **Thermal properties** 22
10. **NMR spectra of the target compounds** 23
11. **References** 34

1. General methods

All starting materials and reagents were purchased from commercial suppliers and used directly without further purification. All solvents for UV and FL analyses were spectroscopic grade. Flash column chromatography was performed on silica gel (200-300 mesh). Thin-layer chromatography (TLC) was performed on precoated silica gel F-254 plates (0.25 mm, E. Merck). ^{1}H NMR and ^{13}C NMR spectra were recorded at r.t. on a Bruker AVANCE III 400 instrument with TMS as an internal reference. EI mass spectra were recorded on the Thermo DSQ mass spectrometer. LC/MS was run on a LCMS-2010A. HRMS were performed on a Thermo MAT95XP mass spectrometer. IR spectra were recorded on an Nicolet Avatar 330 FT-IR. The m.p. values were determined on a WRS-1B digital m.p. apparatus and were not calibrated.

The single crystals were grown from tetrahydrofuran and ethanol (**DF0**, **DF2**, **DF4** and **DF5**), or chloroform and hexane (**DF1**), or chloroform and ethanol (**DF6**). Single-crystal X-ray diffraction data were collected at 293(2) K on an Agilent Technologies Gemini a Ultra system, with Cu-Kα radiation (λ=1.54178 Å**, DF0**, **DF2**, **DF4**, **DF5** and **DF6**) or Mo-Kα radiation (λ=0.7103 Å, **DF1**).

The UV spectra were obtained using a spectrometer (UV-3600 or UV-2700) from Shimadzu. The FL spectra were obtained using a spectrometer (FLS980) from Edinburgh Instruments. The photoluminescence quantum yields were obtained on FLS980 with an integrating sphere (φ 150 mm).

Differential scanning calorimetry (DSC) curves were determined on a NETZSCH DSC 204 F1 instrument under nitrogen at a heating rate of 10 °C/min. Thermogravimetric analyses (TGA) were performed on a NETZSCH TG 209 F3 Tarsus under nitrogen at a heating rate of 10 °C/min.

2. Synthesis of the compound DF

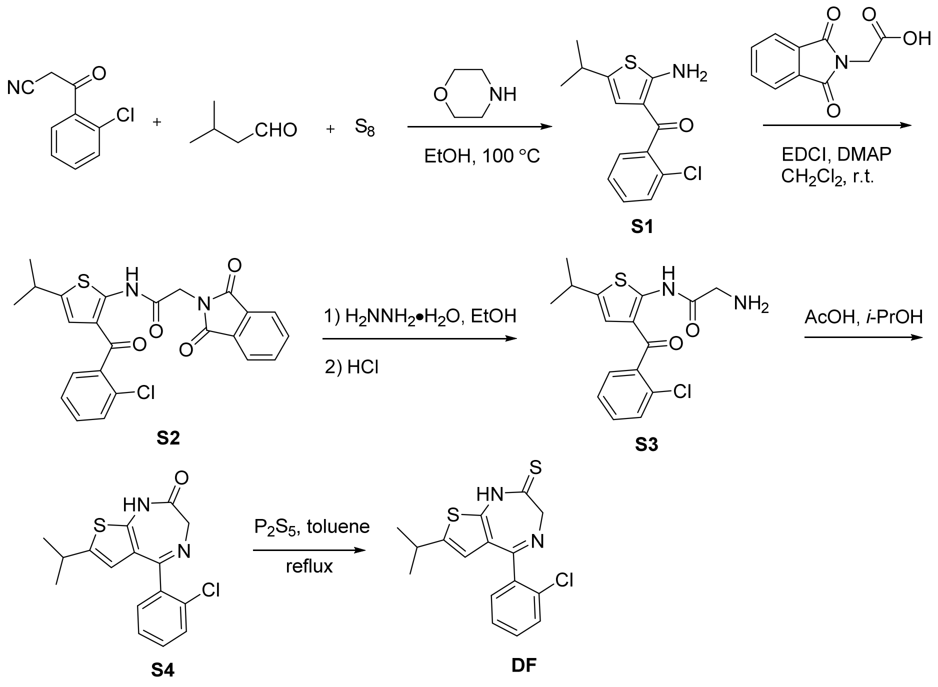

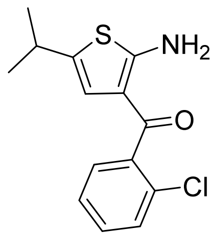

(2-amino-5-isopropylthiophen-3-yl)(2-chlorophenyl)methanone (**S1**) [1]

A 30 mL of vial was added with 3-(2-chlorophenyl)-3-oxopropanenitrile (1.8 g, 10 mmol), S (336 mg, 10.5 mmol), isovaleraldehyde (903 mg, 10.5 mmol), morpholine (870 mg, 10 mmol), EtOH (10 mL) and sealed. The reaction was stirred in an oil bath at 100 °C for 8h. After cooled to room temperature, the mixture was diluted by 250 mL EtOAc. The organic phase was washed with water (40 mL × 3), brine, dried over anhydrous Na_2SO_4, and concentrated in vacuo. The residue was purified by flash c.c. on silica gel to afford **S1**, yellow oil (2.38 g, 85%).

MS (ESI$^+$) m/z: 302 (M+Na$^+$), 334 (M+MeOH+Na$^+$). ^{1}H NMR (400 MHz, $CDCl_3$) δ 7.49 – 7.33 (m, 4H, ArH), 6.10 (s, 1H, ArH), 2.93 – 2.80 (m, 1H, CH), 1.20 (d, *J* = 6.8 Hz, 6H, CH_3). ^{13}C NMR (101 MHz, $CDCl_3$) δ 188.7, 165.5, 140.4, 133.6, 130.5, 130.2, 129.9, 128.4, 126.6, 120.1, 114.9, 29.6, 24.0.

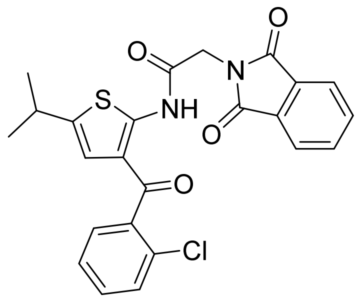

N-(3-(2-chlorobenzoyl)-5-isopropylthiophen-2-yl)-2-(1,3-dioxoisoindolin-2-yl)acetamide (**S2**) [1]

To a 100 mL round flask was added **S1** (1.68 g, 6 mmol), *N*-phthaloylglycine (2.05 g, 10 mmol), dimethyllaminopropyl)carbodiimide hydrochloride (2.3 g, 12 mmol), 4-dimethylaminopyridine (1.46 g, 12 mmol), dichloromethane and a magnetic stir bar. The reaction mixture was stirred at r.t. over night. After completation of the reaction, the mixture was filtered and the residue was washed with CH_2Cl_2 (10 mL × 3). The filtrate was concentrated in vacuo to remove the solvent, then the residue was crystallized from MeOH to afford **S2**, white solids (2.52 g, 90%).

MS (ESI$^+$) m/z: 489 (M+Na$^+$), 521 (M+MeOH+Na$^+$). ^{1}H NMR (400 MHz, $CDCl_3$) δ 12.25 (brs, 1H, NH), 7.99 – 7.90 (m, 2H, ArH), 7.82 – 7.75 (m, 2H, ArH), 7.51 – 7.34 (m, 4H, ArH), 6.38 (s, 1H, ArH), 4.73 (s, 2H. CH_2), 3.06 – 2.94 (m, 1H, CH), 1.25 (d, *J* = 6.8 Hz, 6H, CH_3). ^{13}C NMR (101 MHz, $CDCl_3$) δ 191.4, 167.5, 164.2, 149.0, 143.2, 139.1, 134.3, 132.0, 131.0, 130.6, 130.2, 128.4, 126.7, 123.8, 120.9, 119.6, 40.8, 29.5, 24.3.

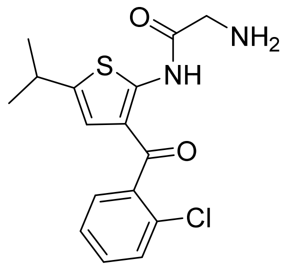

2-amino-*N*-(3-(2-chlorobenzoyl)-5-isopropylthiophen-2-yl)acetamide (**S3**) [1]

In a 250 mL round bottom flask, **S2** (2.33 g, 5 mmol) was dispersed in 150 mL MeOH and cooled by an ice-bath. Hydrazine hydrate (80%, 10 mmol) was added and the reaction was stirred for 8 h then allowed to warm to r.t.. Hydrochloric acid (36%, 20 mmol) was added and the reaction mixture stirred at r.t. over night. The insoluble solids were filtered off and the filtrate was neutralized with saturated aqueous $NaHCO_3$. After removal of solvent, 200 mL CH_2Cl_2 was added to the residue and the organic phase was washed with water (40 mL × 3), brine, dried over anhydrous Na_2SO_4, and concentrated in vacuo. The residue was crystallized from hexane to afford **S3**, pale yellow solids (1.48 g, 88%).

MS (ESI$^+$) m/z: 337 (M+H$^+$), 359 (M+Na$^+$), 391 (M+MeOH+Na$^+$). ^{1}H NMR (400 MHz, $CDCl_3$) δ 12.88 (brs, 1H, NH), 7.50 – 7.36 (m, 4H, ArH), 6.39 (s, 1H, ArH), 3.71 (s, 2H, CH_2), 3.06 – 2.97 (m, 1H, CH), 2.07 (brs, 2H, NH_2), 1.28 (d, J = 6.8 Hz, 6H, CH_3). ^{13}C NMR (101 MHz, $CDCl_3$) δ 190.7, 171.7, 148.9, 142.4, 139.6, 130.8, 130.5, 130.0, 128.4, 126.7, 120.7, 119.7, 44.8, 29.5, 24.3.

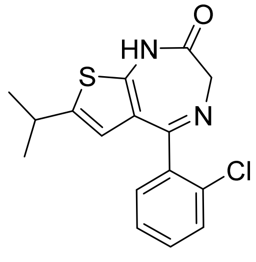

5-(2-chlorophenyl)-7-isopropyl-1,3-dihydro-2*H*-thieno[2,3-e][1,4]diazepin-2-one (**S4**) [1]

A 30 mL of vial was added with **S3** (1.35 g, 4 mmol), AcOH (800 mg), isopropyl alcohol (6 mL) and sealed. The reaction was stirred in an oil bath at 70 ℃ for 8 h. After cooled to room temperature, 150 mL CH_2Cl_2 was added and the organic solvent was washed with saturated aqueous $NaHCO_3$ (40 mL × 3), water, brine, dried over anhydrous Na_2SO_4, and concentrated in vacuo. The residue was crystallized from EtOAc to afford **S4**, pale yellow solid (765 mg, 60%).

MS (ESI$^+$) m/z: 319 (M+H$^+$), 341 (M+Na$^+$), 373 (M+MeOH+Na$^+$). ^{1}H NMR (400 MHz, $CDCl_3$) δ 10.25 (brs, 1H, NH), 7.47 – 7.32 (m, 4H, ArH), 6.21 (s, 1H, ArH), 4.51 (s, 2H, CH_2), 3.07 – 2.93 (m, 1H, CH), 1.25 (d, J = 6.8 Hz, 6H, CH_3). ^{13}C NMR (101 MHz, $CDCl_3$) δ 168.3, 166.7, 145.2, 142.7, 138.3, 133.0, 130.7, 130.5, 129.9, 126.8, 126.3, 120.2, 57.4, 29.9, 24.3.

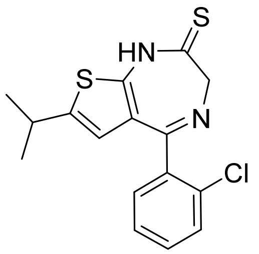

5-(2-chlorophenyl)-7-isopropyl-1,3-dihydro-2*H*-thieno[2,3-e][1,4]diazepine-2-thione (**DF**) [1]

S4 (638 mg, 2 mmol), phosphorus pent sulfide (3 g) and toluene (50 mL) were added to a three necks round bottom flask under nitrogen atmosphere. The mixture was stirred under reflux for 12 h. After removal of solvent, 100 mL saturated aqueous $NaHCO_3$ was added to the residue and the mixture was stirred over night. **DF** was obtained after filtrate and washed with water, yellow solid (600 mg, 90%);

MS (ESI$^+$) m/z: 335 (M+H$^+$), 357 (M+Na$^+$), 389 (M+MeOH+Na$^+$). ^{1}H NMR (400 MHz, $CDCl_3$) δ 7.55 – 7.32 (m, 4H, ArH), 6.25 (s, 1H, ArH), 4.93 (s, 2H, CH_2), 3.07 – 2.95 (m, 1H, CH), 1.26 (d, J = 6.8 Hz, 6H, CH_3). ^{13}C NMR (101 MHz, $CDCl_3$ and DMSO-d_6) δ 199.2, 170.8, 150.5, 149.9, 142.5, 137.5, 135.6, 135.4, 134.6, 132.5, 131.6, 125.1, 69.9, 34.5, 29.0.

3. General Procedure for syntheses of target compounds

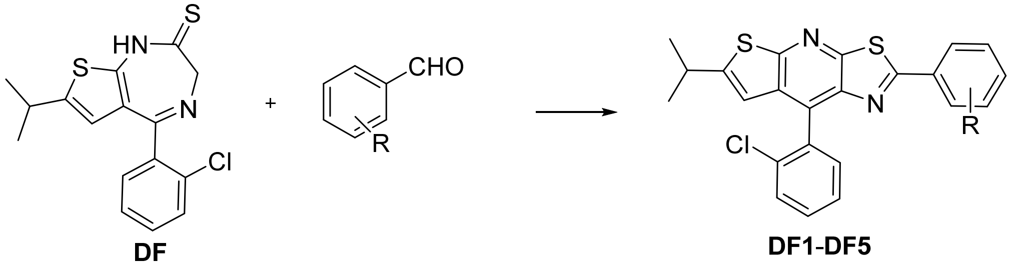

A 10 mL of vial was added with compound 5-(2-chlorophenyl)-7-isopropyl-1,3-dihydro-2H-thieno[2,3-e][1,4]diazepine-2-thione (**DF**, 100 mg, 0.3 mmol), $Sc(OTf)_3$ (1.5 mg, 0.003 mmol), corresponding aldehyde (0.6 mmol), NMP (2.0 mL) and sealed. The reaction was stirred in an oil bath preheated to 150 °C for 12 h. After cooled to room temperature, the reaction mixture was dilute with 120 mL EtOAc. The organic phase was washed with saturated aqueous NH_4Cl (30 mL × 3), water and brine, dried over anhydrous Na_2SO_4, and concentrated in vacuo. The residue was purified by flash c.c. on silica gel to afford desired compound. [1]

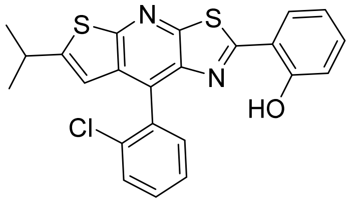

5-(2-chlorophenyl)-7-isopropyl-1,3-dihydro-2*H*-thieno[2,3-e][1,4]diazepine-2-thione (**DF1**)

Yellow solids (63 mg, 51%); m.p.: 192-193 °C; MS (EI): m/z (%) = 436 (95) [M$^+$], 438 (40), 421 (100), 401 (50). HRMS–EI: m/z M$^+$ calcd for $C_{23}H_{17}O_1N_2ClS_2$: 436.0465; found: 436.0464. ^{1}H NMR (400 MHz, $CDCl_3$): δ = 12.09

(brs, 1H, OH), 7.71-7.67 (m, 2H, ArH), 7.55-7.48 (m, 3H, ArH), 7.40-7.36 (m, 1H, ArH), 7.04-6.95 (m, 2H, ArH), 6.81 (s, 1H, ArH), 3.34 – 3.18 (m, 1H, CH, CH), 1.42 (d, J = 6.9 Hz, 6H, CH_3). ^{13}C NMR (100 MHz, $CDCl_3$): δ = 168.6, 159.7, 158.2, 155.4, 153.0, 140.9, 133.9, 133.3, 133.2, 133.1, 131.5, 130.4, 130.2, 128.6, 126.9, 119.7, 117.9, 116.8, 115.2, 31.4, 24.1, 24.0. IR (KBr): 3058, 2961, 2927, 2868, 1620, 1581, 1548, 1481, 1439, 1426, 1309, 1283, 1250, 1216, 1153, 1050, 1033, 966, 830, 758, 748, 700 cm^{-1}.

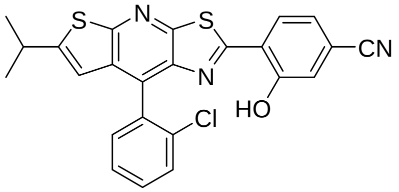

4-(8-(2-chlorophenyl)-6-isopropylthiazolo[5,4-b]thieno[3,2-e]pyridin-2-yl)-3-hydroxybenzonitrile (**DF2**)

Yellow solids (60 mg, 43%); m.p.: 227-229 °C; MS (EI): m/z (%) = 461 (68) [M^+], 463 (28), 446 (100), 426 (40), 206 (60). HRMS–EI: m/z M^+ calcd for $C_{24}H_{16}ON_3ClS_2$: 461.0418; found: 461.0416. ^1H NMR (400 MHz, $CDCl_3$) δ 12.38 (s, 1H, OH), 7.83 – 7.65 (m, 2H, ArH), 7.61 – 7.45 (m, 3H, ArH), 7.34 – 7.19 (m, 2H, ArH), 6.82 (s, 1H, ArH), 3.47 – 3.08 (m, 1H, CH), 1.43 (d, J = 5.6 Hz, 6H, CH_3). ^{13}C NMR (101 MHz, $CDCl_3$) δ 166.5, 160.8, 158.0, 156.2, 152.7, 140.5, 133.8, 133.5, 133.2, 131.9, 131.3, 130.7, 130.3, 129.1, 127.0, 122.6, 121.8, 120.4, 118.0, 115.6, 115.1, 31.4, 24.0, 24.0. IR (KBr): 3432, 3046, 2963, 2924, 2867, 1611, 1568, 1546, 1467, 1366, 1313, 1279, 1244, 1208, 1109, 1087, 1051, 971, 825, 772, 752, 720 cm^{-1}.

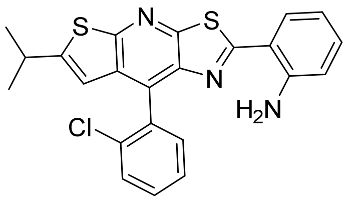

2-(8-(2-chlorophenyl)-6-isopropylthiazolo[5,4-b]thieno[3,2-e]pyridin-2-yl)aniline (**DF3**)

Yellow solids (33 mg, 25%); m.p.: 115-116 °C; MS (EI): m/z (%) = 435 (100) [M^+], 437 (40), 420 (95), 400 (40). HRMS–EI: m/z M^+ calcd for $C_{23}H_{18}N_3ClS_2$: 435.0625; found: 435.0623. ^1H NMR (400 MHz, $CDCl_3$): δ = 7.72-7.70 (m, 1H, ArH), 7.65-7.62 (m, 1H, ArH), 7.56-7.54 (m, 1H, ArH), 7.51-7.44 (m, 2H, ArH), 7.24-7.20 (m, 1H, ArH), 6.77-6.71 (m, 3H, ArH), 3.24 (m, 1H, CH), 1.42 (d, J = 6.8 Hz, 6H, CH_3). ^{13}C NMR (100 MHz, $CDCl_3$): δ = 168.2, 158.9, 154.5, 154.1, 146.9, 142.6, 134.7, 133.4, 133.0, 131.9, 131.7, 131.1, 130.4, 129.9, 126.7, 117.0, 116.9, 115.2, 115.1, 31.3, 24.1, 24.0. IR (KBr): 3471, 3443, 3315, 2963, 2924, 2868, 1615, 1588, 1494, 1470, 1442, 1320, 1312, 1278, 1211, 1159, 1048, 1033, 956, 826, 750, 734, 704 cm^{-1}.

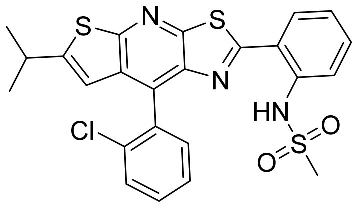

N-(2-(8-(2-chlorophenyl)-6-isopropylthiazolo[5,4-b]thieno[3,2-e]pyridin-2-yl)phenyl)methanesulfonamide (**DF4**)

Yellow solids (60 mg, 39%); m.p.: 189-190 °C; MS (EI): m/z (%) = 513 (100) [M^+], 515 (45), 498(50), 434 (95), 418 (80), 382(50). HRMS–EI: m/z M^+ calcd for $C_{24}H_{20}N_3ClO_2S_3$: 513.0401; found: 513.0394. ^{1}H NMR (400 MHz, $CDCl_3$): δ = 11.45 (brs, 1H, NH), 7.88-7.83 (m, 2H, ArH), 7.71-7.70 (m, 1H, ArH), 7.56-7.47 (m, 4H, ArH), 7.22-7.21 (m, 1H, ArH), 6.81 (s, 1H, ArH), 3.25 (m, 1H, CH), 2.91 (s, 3H, CH_3), 1.42 (d, J = 6.7 Hz, 6H, CH_3). ^{13}C NMR (100 MHz, $CDCl_3$) δ 167.2, 160.3, 155.5, 153.2, 141.7, 137.7, 134.2, 133.6, 133.0, 132.5, 131.7, 131.0, 130.8, 130.6, 130.3, 127.3, 123.5, 119.4, 119.0, 115.2, 39.8, 31.4, 24.1, 24.0. IR (KBr): 3055, 3010, 2963, 2924, 2868, 2851, 1578, 1549, 1484, 1471, 1430, 1342, 1312, 1280, 1240, 1204, 1155, 1088, 1052, 961, 901, 793, 754, 733, 703, 533, 511 cm^{-1}.

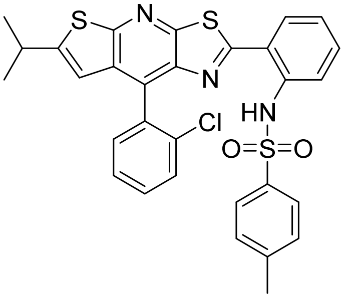

N-(2-(8-(2-chlorophenyl)-6-isopropylthiazolo[5,4-b]thieno[3,2-e]pyridin-2-yl)phenyl)-4-methylbenzenesulfonamide (**DF5**)

Pale yellow solids (80 mg, 45%); m.p.:194-196 °C; MS (EI): m/z (%) = 589 (62) [M^+], 591 (38), 434 (100), 418 (80), 382 (35), 91 (95). HRMS–EI: m/z M^+ calcd for $C_{30}H_{24}N_3ClO_2S_3$: 589.0714; found: 589.0719. ^{1}H NMR (400 MHz, $CDCl_3$) δ = 11.63 (brs, 1H, NH), 7.89 – 7.68 (m, 3H, ArH), 7.61 (m, 3H. ArH), 7.47 – 7.32 (m, 3H, ArH), 7.16 – 7.02 (m, 3H, ArH), 6.84 (s, 1H, ArH), 3.35 – 3.19 (m, 1H, CH), 2.29 (s, 3H, CH_3), 1.43 (d, J = 6.6 Hz, 6H, CH_3). ^{13}C NMR (101 MHz, $CDCl_3$) δ = 167.2, 160.1, 155.4, 153.3, 143.5, 141.7, 137.4, 136.7, 134.2, 133.8, 133.0, 132.1, 131.7, 131.1, 130.7, 130.5, 130.3, 129.4, 127.5, 127.0, 123.4, 119.8, 119.6, 115.3, 31.4, 24.1, 24.0, 21.5. IR (KBr): 3429, 3056, 2962, 2922, 1601, 1578, 1550, 1505, 1484, 1431, 1350, 1313, 1282, 1209, 1165, 1123, 1091, 915, 809, 758, 737, 704, 666, 626, 561 cm^{-1}.

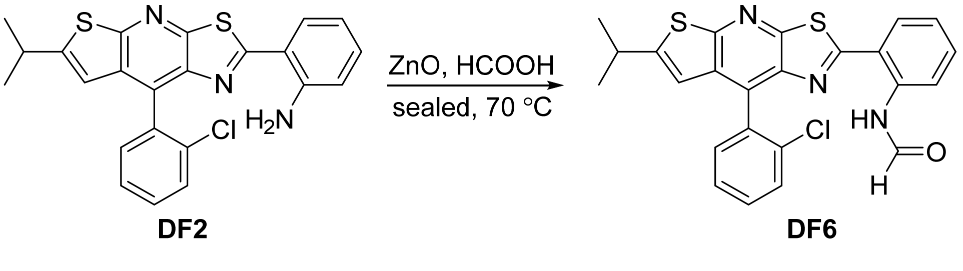

N-(2-(8-(2-chlorophenyl)-6-isopropylthiazolo[5,4-b]thieno[3,2-e]pyridin-2-yl)phenyl)formamide (**DF6**)

A 10 mL of vial was added with compound 2-(8-(2-chlorophenyl)-6-isopropylthiazolo[5,4-b]thieno[3,2-e]pyridin-2-yl)aniline (**DF2**, 88 mg, 0.2 mmol), ZnO (8 mg, 0.1 mmol), HCOOH (1.0 mL) and sealed. The reaction was stirred in an oil bath preheated to 70 °C for 6 h. After cooled to room temperature, the reaction mixture was dilute with 120 mL EtOAc. The organic phase was washed with saturated aqueous $NaHCO_3$ (30 mL × 3), water and brine, dried over anhydrous Na_2SO_4, and concentrated in vacuo. The residue was purified by flash c.c. on silica gel to afford

DF6, pale yellow solids (46 mg, 50%); m.p.: 234-236 °C; MS (EI): m/z (%) = 463 (20) [M^+], 465 (7) [M^+], 435 (100), 437 (42), 420 (70), 422 (30). HRMS–EI: m/z M^+ calcd for $C_{24}H_{18}ON_3ClS_2$: 463.0574; found: 463.0578.

^{1}H NMR (400 MHz, $CDCl_3$) δ 12.14 (s, 0.9H. CHO), 11.35 (s, 0.1H, CHO), 8.96 (d, *J* = 10.3 Hz, 0.1H, ArH), 8.75 (d, *J* = 8.4 Hz, 0.9H, ArH), 8.20 (s, 0.9H, NH), 7.87 (d, *J* = 7.8 Hz, 1H, ArH), 7.76 – 7.65 (m, 1H, ArH), 7.60 – 7.41 (m, 4H, ArH), 7.27 – 7.17 (m, 1H, ArH), 6.85 (s, 1H, ArH), 3.35 – 3.20 (m, 1H, CH), 1.43 (d, *J* = 6.8 Hz, 6H, CH_3). ^{13}C NMR (101 MHz, $CDCl_3$) δ 167.5, 160.2, 159.4, 155.6, 153.3, 141.7, 137.4, 134.3, 133.5, 133.4, 132.4, 131.6, 131.4, 130.4, 130.0, 130.0, 126.9, 123.8, 121.6, 119.0, 115.0, 31.4, 24.1, 24.0. IR (KBr): 3450, 3187, 3110, 2963, 2859, 2775, 1702, 1587, 1532, 1491, 1438, 1293, 1254, 1212, 1152, 1106, 1049, 1029, 957, 847, 803, 752, 702, 626, 517 cm^{-1}.

4. Preparation of room temperature glasses

Dispersions of **DF5** in room-temperature glasses of sucrose octaacetate (SOA) were prepared by melting approximately 5 g of purified SOA powder over gentle heat in a glass beaker, adding a measured solution of **DF5** solution (50 µL of a 1 mM stock solution in THF), and stirring gently to disperse **DF5**, The melt was transferred to a quartz slide, where it quickly hardened into a transparent, glassy solid. [2]

5. Properties of the single crystal structures

Table S1: Crystal data and structure refinement for the single crystals.

compounds	DF0	DF1	DF2	DF4	DF5	DF6
formula	$C_{23}H_{17}N_2OClS_2·EtOH·H_2O$	$C_{23}H_{17}N_2OClS_2$	$C_{24}H_{16}ClN_3OS_2$	$C_{24}H_{20}N_3O_2ClS_3$	$C_{30}H_{24}N_3O_2ClS_3$	$C_{24}H_{18}N_3OClS_2$
fw	501.04	436.97	461.98	514.07	590.17	463.98
crystal system	triclinic	orthorhombic	monoclinic	triclinic	triclinic	Monoclinic
T (K)	293 (2)	293 (2)	293 (2)	293 (2)	293(2)	293(2)
space group	P1	Pbca	P2(1)/c	P1	P1	P2(1)/c
a / Å	8.3113(5)	9.1702(4)	14.1636(4)	9.5948(6)	10.6742(5)	13.6964(2)
b / Å	12.1970(6)	18.4101(9)	7.6109(2)	10.3128(7)	10.9730(6)	8.14000(10)
c / Å	12.7574(7)	24.3386(11)	20.6614(5)	12.0188(8)	13.2942(7)	20.1669(2)
α / °	101.086(4)	90.00	90	87.285(5)	112.061(5)	90
β / °	105.393(5)	90.00	103.485(3)	86.081(5)	95.450(4)	106.8190(10)
γ / °	93.456(5)	90.00	90	83.966(5)	93.025(4)	90
V/Å^3, Z	1215.10(12), 2	4108.9(3), 8	2165.85(10), 4	1178.93(13), 2	1429.98(13), 2	2152.20(5), 4
F (000)	524	1808	952	532	612	960
crystal size / mm^3	0.42×0.37×0.32	0.1×0.1×0.1	0.18×0.16×0.13	0.15×0.12×0.1	0.18×0.15×0.12	0.35×0.27×0.12
reflections collected / unique (R_{int})	8606 / 4235 (R_{int} = 0.0343)	17973 / 5086 (R_{int} = 0.0342)	7154 / 3271 (R_{int} = 0.0276)	6797 / 3550 (R_{int} = 0.0307)	8252 / 4301 (R_{int} = 0.0242)	7876 / 4022 (R_{int} = 0.0586)
data / restraints /parameter	4235/0/303	5086/6/274	3271/24/294	3550/1/266	4301/0/356	4022/0/286
D_c / mg·m^{-3}	1.369	1.413	1.417	1.448	1.371	1.432
μ / mm^{-1}	3.243	0.407	3.541	4.147	3.495	3.563
goodness-of-fit on F^2	1.028	1.029	1.029	1.046	1.030	1.060
R_1,[a] wR_2[b] [I≥2σ(I)]	0.0507, 0.1294	0.0627, 0.1646	0.0444, 0.1225	0.0651, 0.1708	0.0574, 0.1593	0.0607, 0.1655
R_1,[a] wR_2[b] (all data)	0.0609, 0.1393	0.0833, 0.1821	0.0536, 0.1325	0.0756, 0.1820	0.0680, 0.1711	0.0642, 0.1727

[a] $R_1 = \Sigma||F_o|-|F_c||/\Sigma|F_o|$. [b] $wR_2 = [\Sigma[w(F_o^2 - F_c^2)^2]/\Sigma w(F_o^2)^2]^{1/2}$, where $w = 1/[\sigma^2(F_o)^2 + (aP)^2 + bP]$ and $P = (F_o^2 + 2F_c^2)/3$.

CCDC 1435784 (**DF0**), CCDC 1435785 (**DF1**), CCDC 1435786 (**DF2**), CCDC 1435787 (**DF4**), CCDC 1435788 (**DF5**) and CCDC 1457981 (**DF6**) contain the supplementary crystallographic data for this paper. These data can be obtained free of charge from The Cambridge Crystallographic Data Center via www.ccdc.cam.ac.uk/data_request/cif.

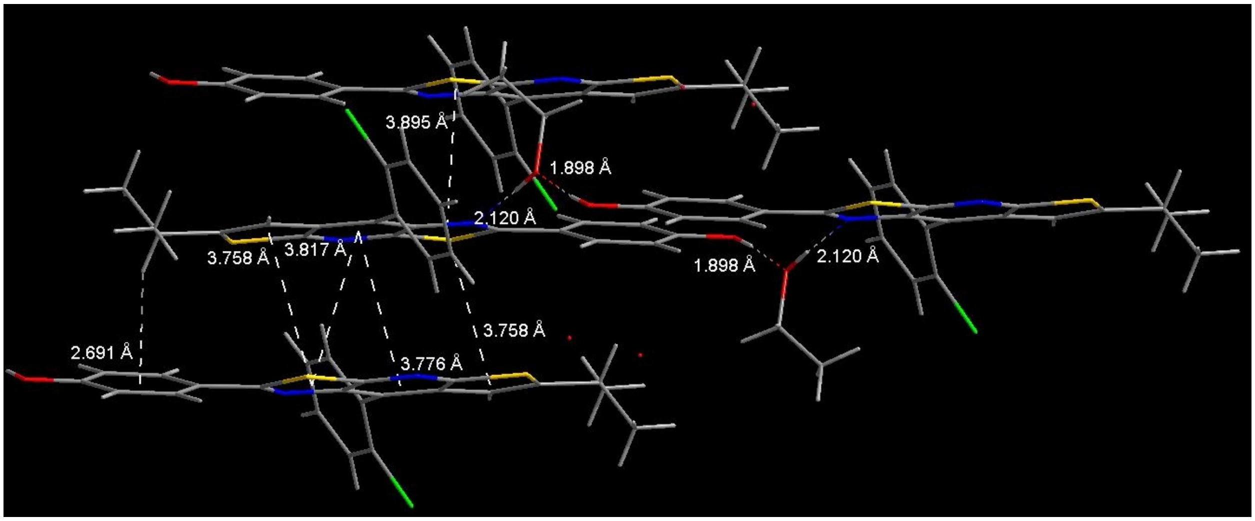

Figure S1: intermolecular interactions of DF0 in single crystals

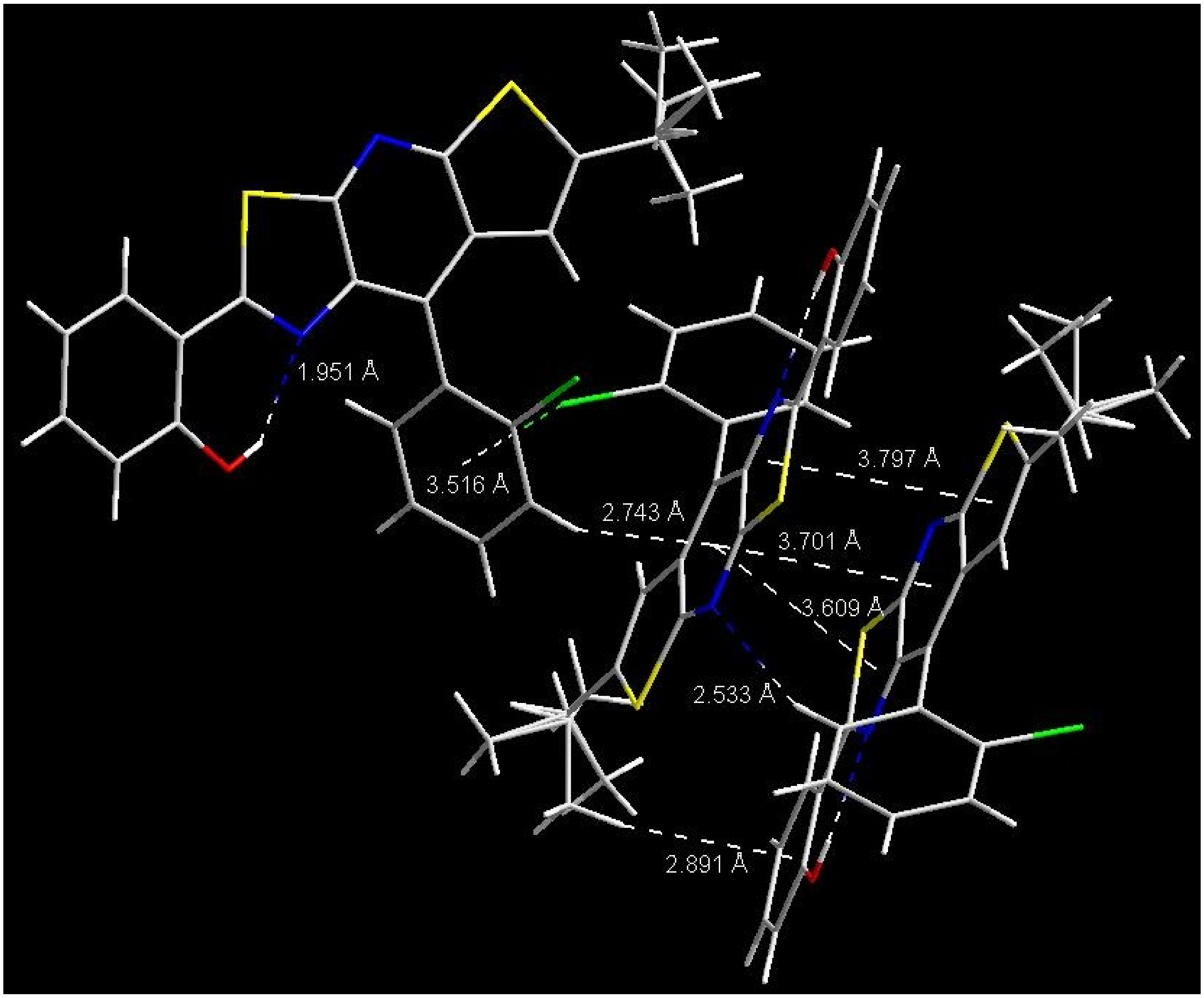

Figure S2: intermolecular and intramolecular interactions of DF1 in single crystals

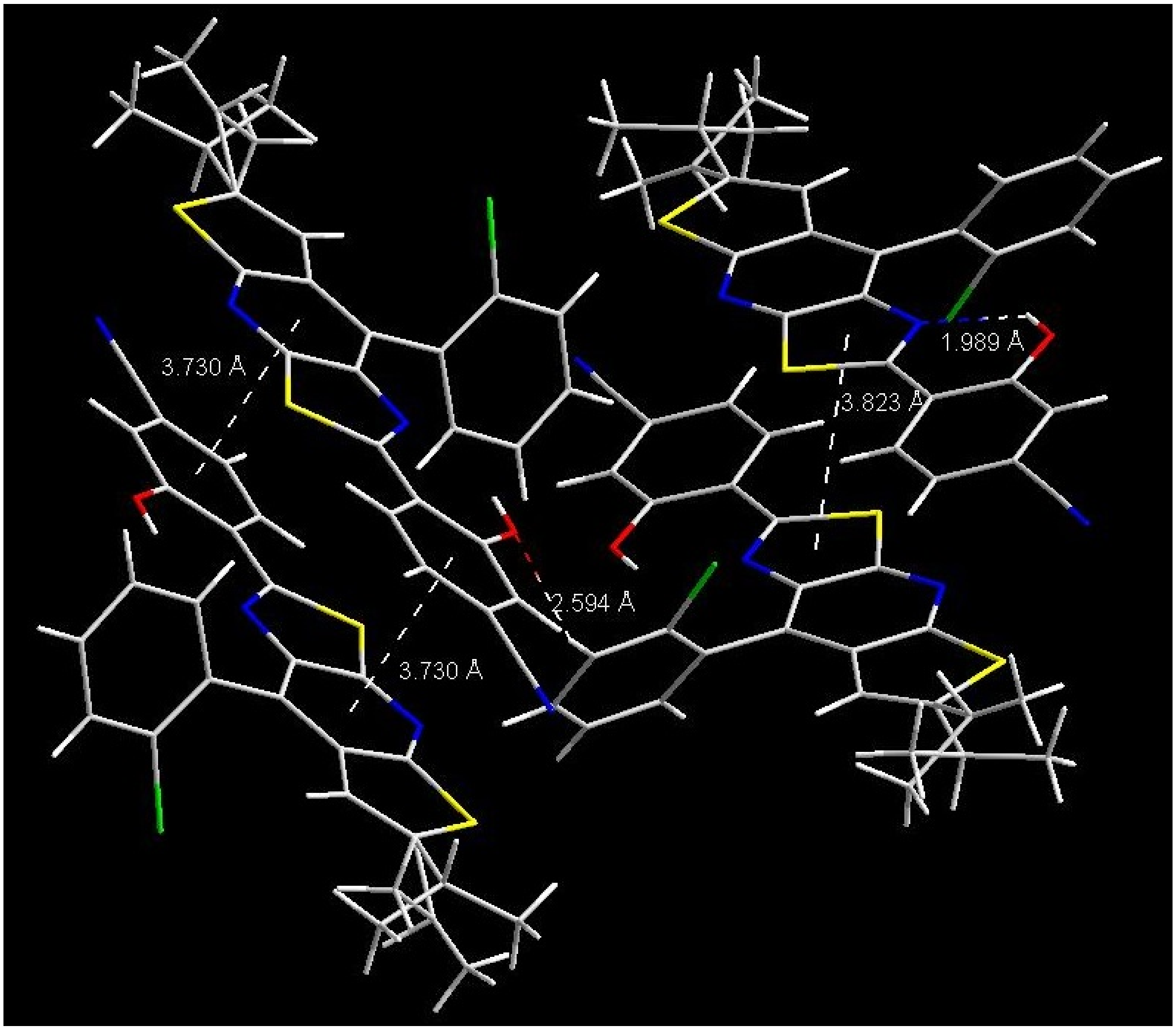

Figure S3: intermolecular and intramolecular interactions of DF2 in single crystals

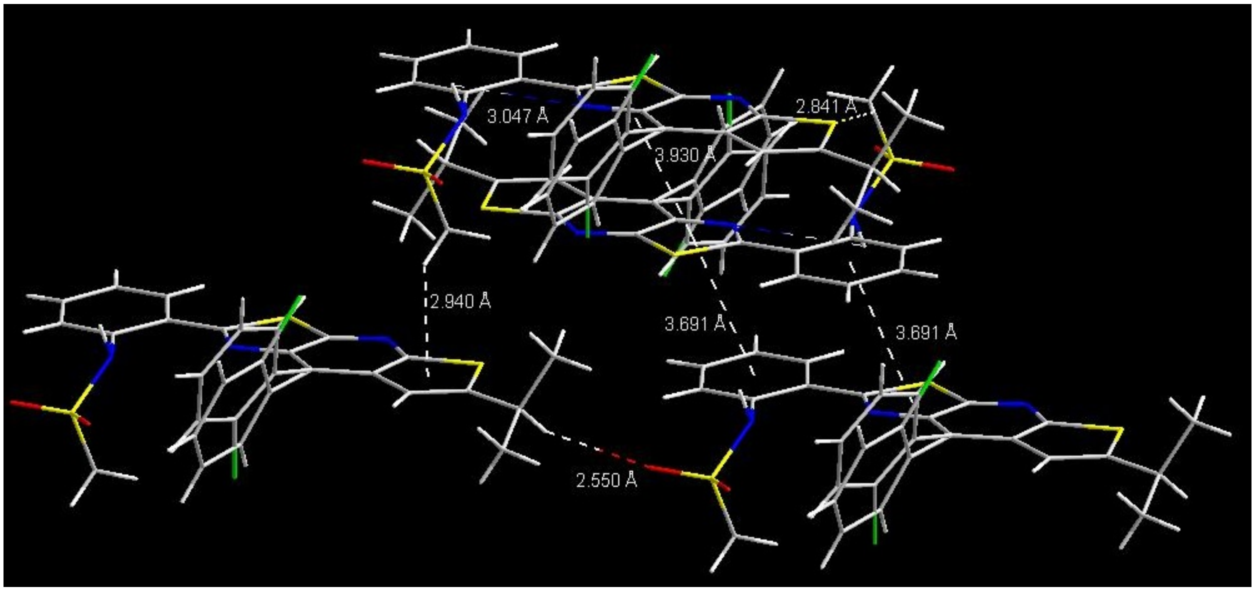

Figure S4: intermolecular and intramolecular interactions of DF4 in single crystals

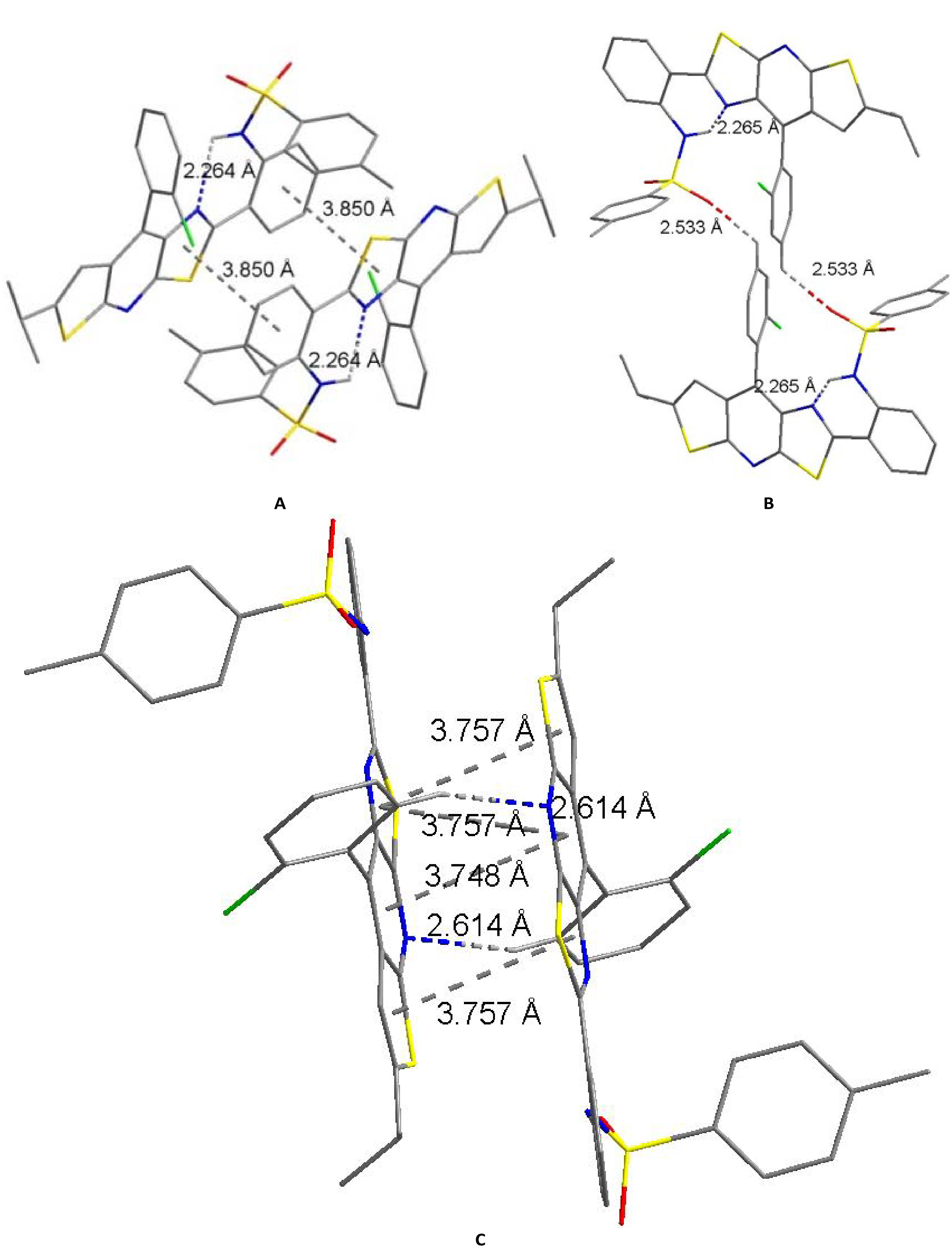

Figure S5: intermolecular and intramolecular interactions of DF5 in single crystals

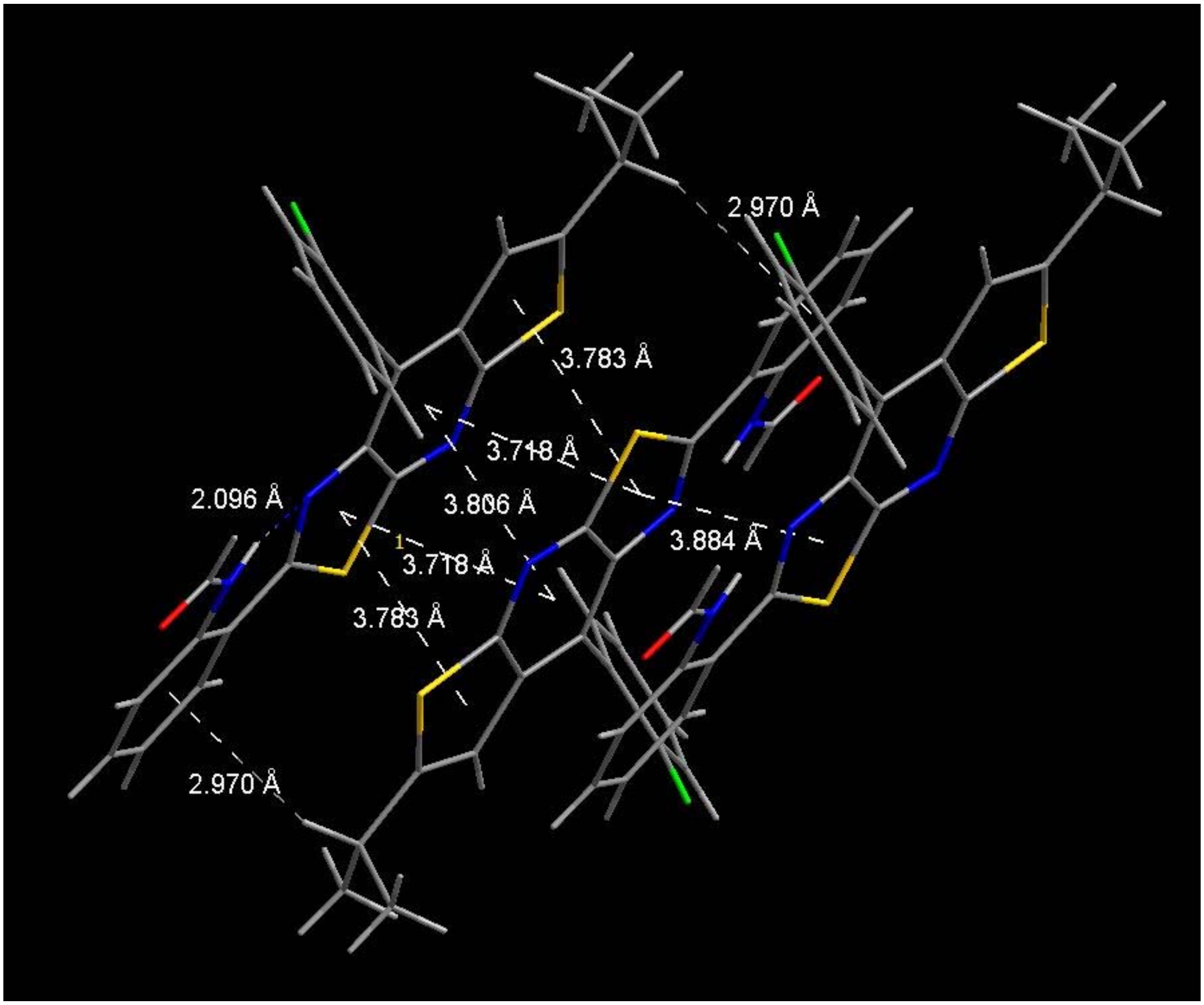

Figure S6: intermolecular and intramolecular interactions of DF6 in single crystals

6. UV absorption spectra of the target compounds

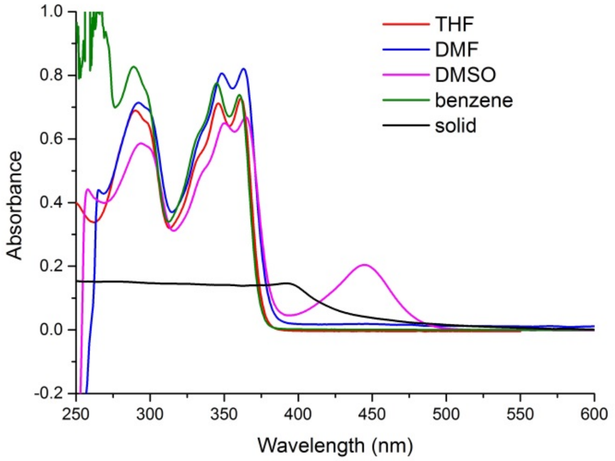

DF0

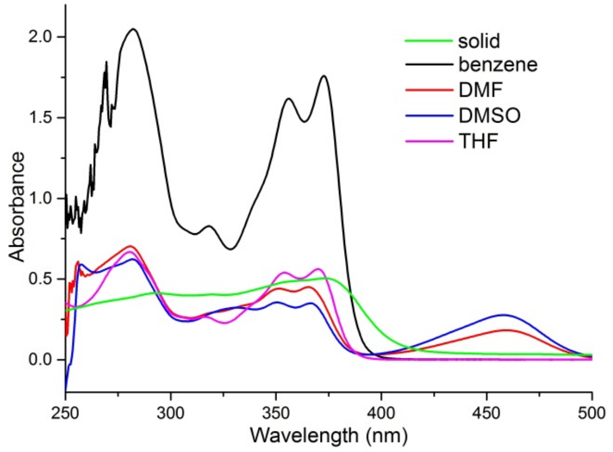

DF1

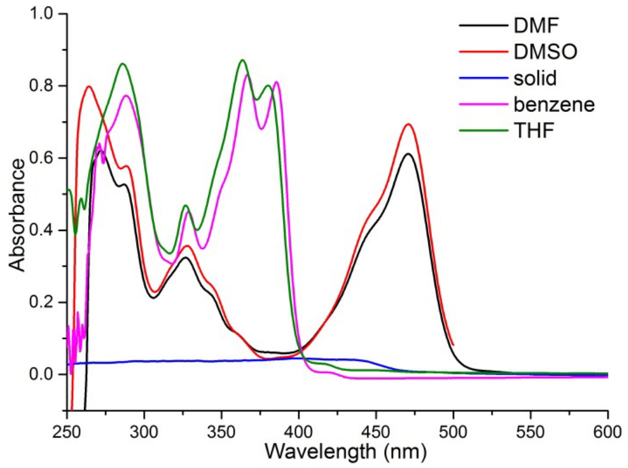

DF2

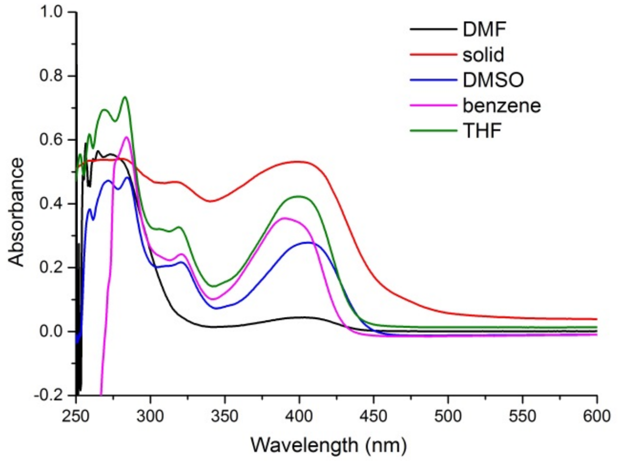

DF3

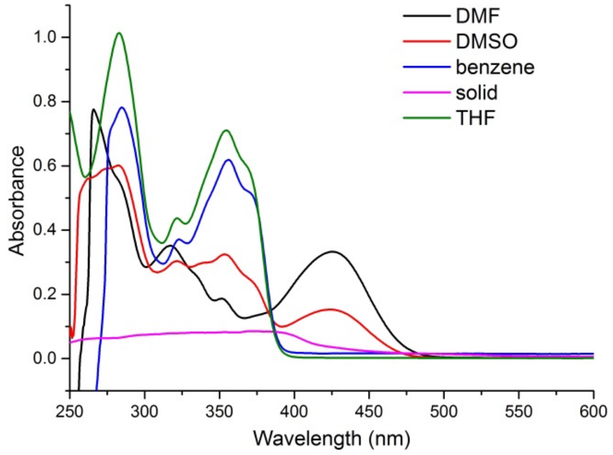

DF4

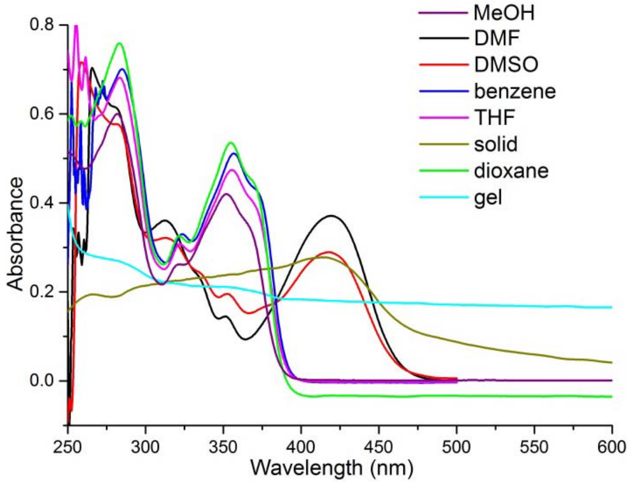

DF5

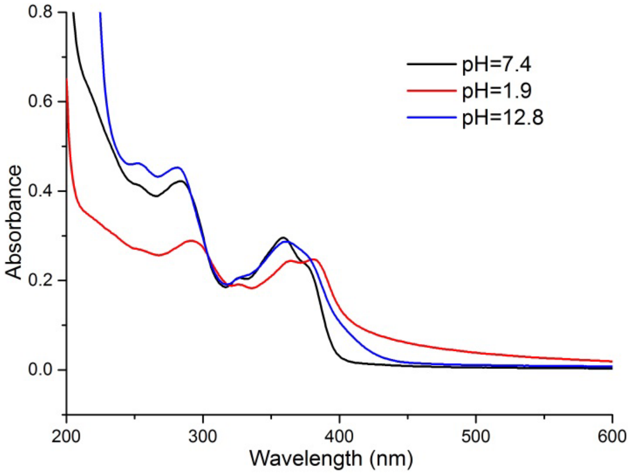

DF5

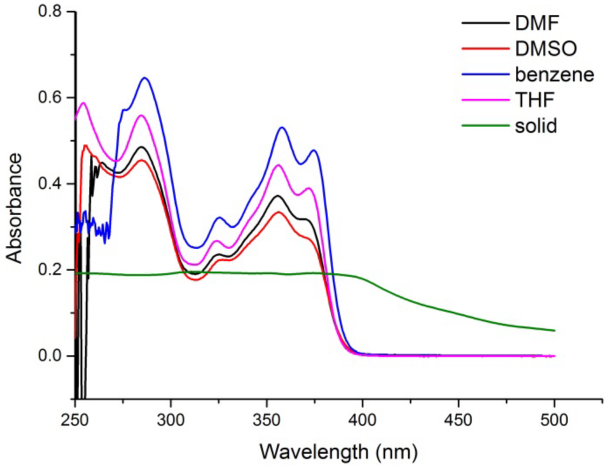

DF6

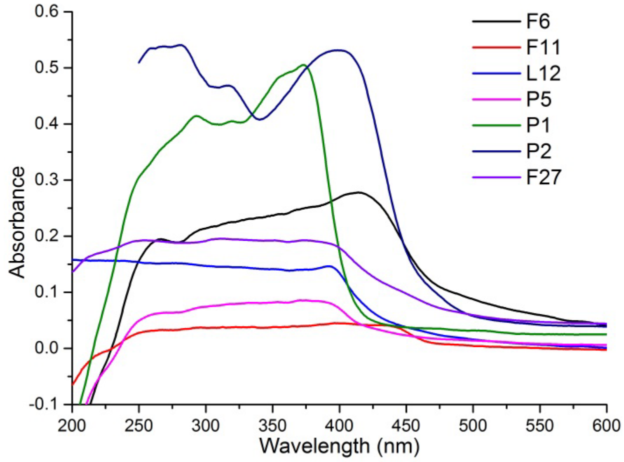

Solid state

Figure S7: UV absorption spectra of compounds in different solvents (2×10^{-5} M) and solid state.

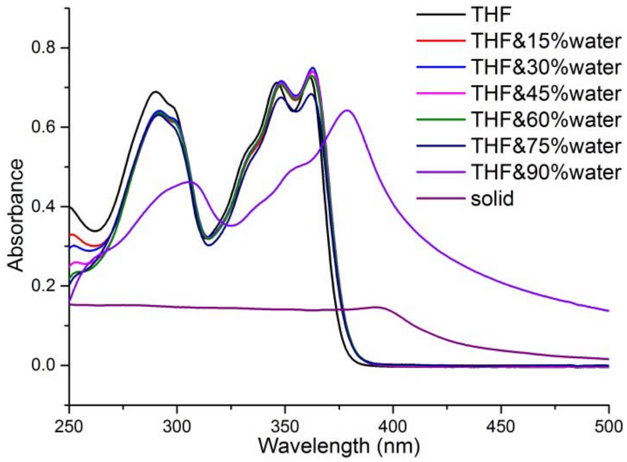

DF0

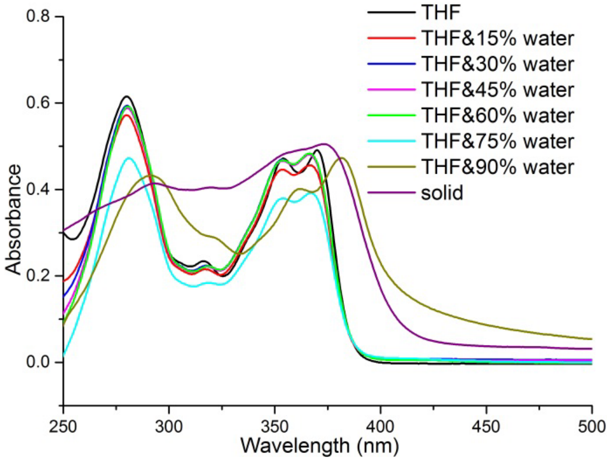

DF1

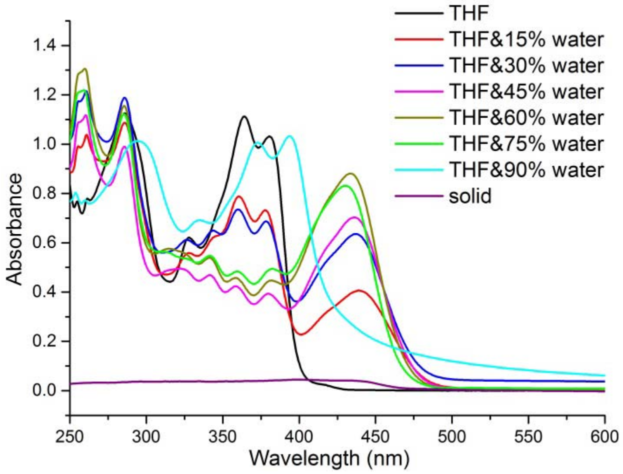

DF2

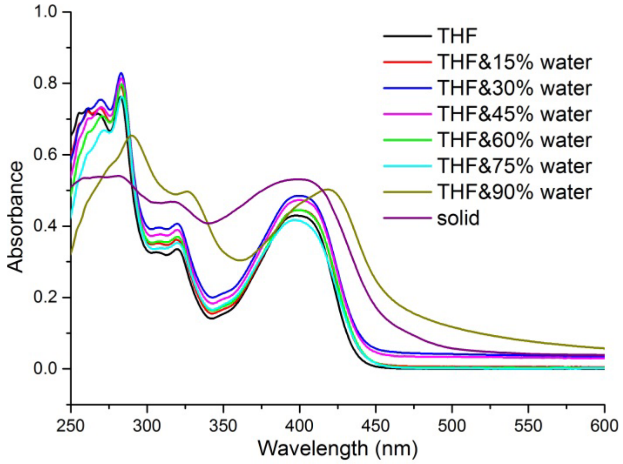

DF3

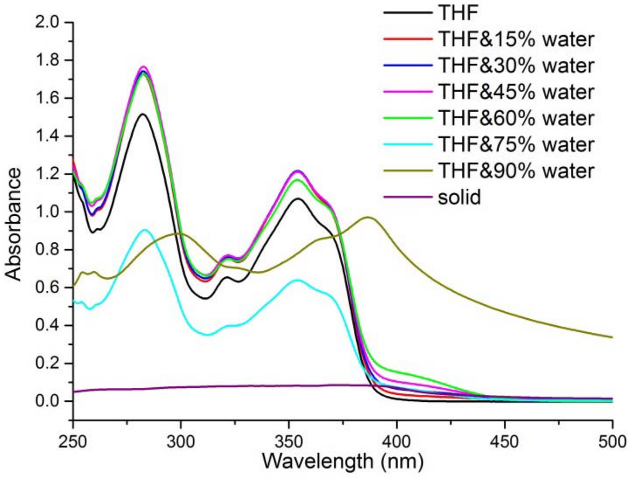

DF4

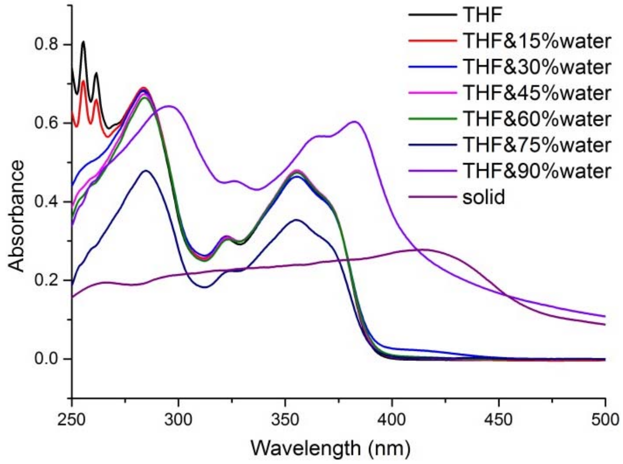

DF5

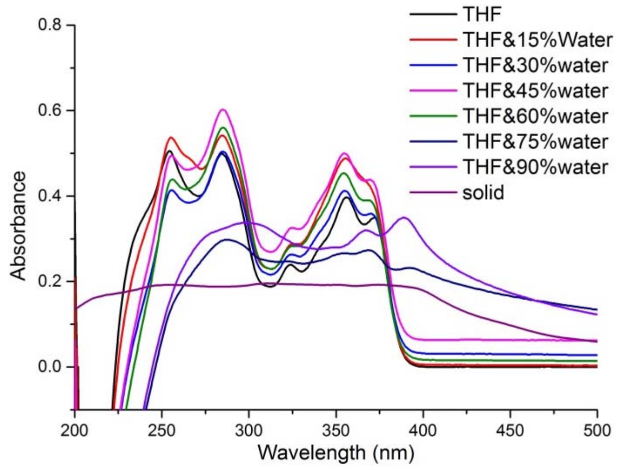

DF6

Figure S8: UV absorption spectra of compounds in THF/water mixtures with different amounts of water (2×10^{-5} M) and solid state.

7. Excitation spectra of the target compounds

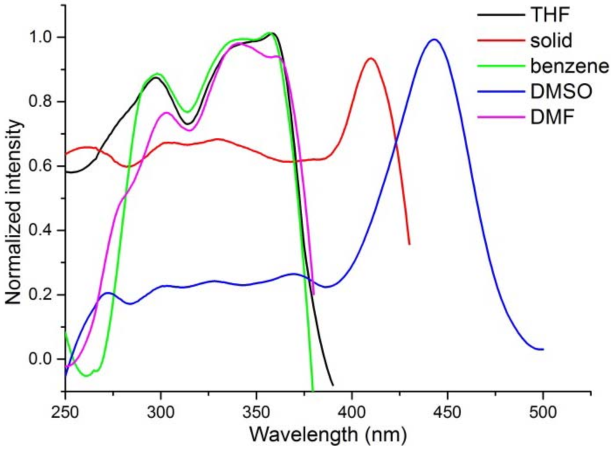

DF0

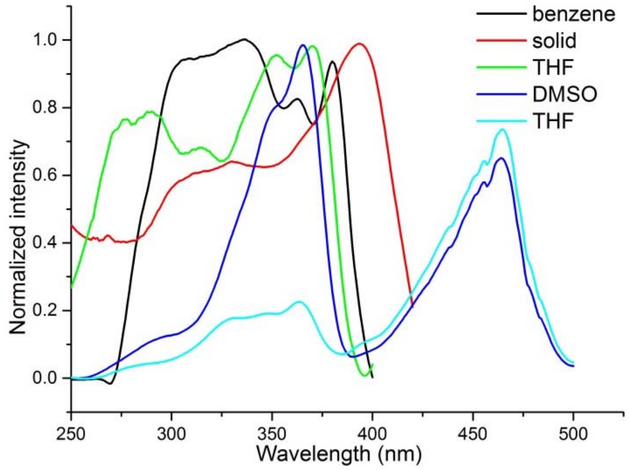

DF1

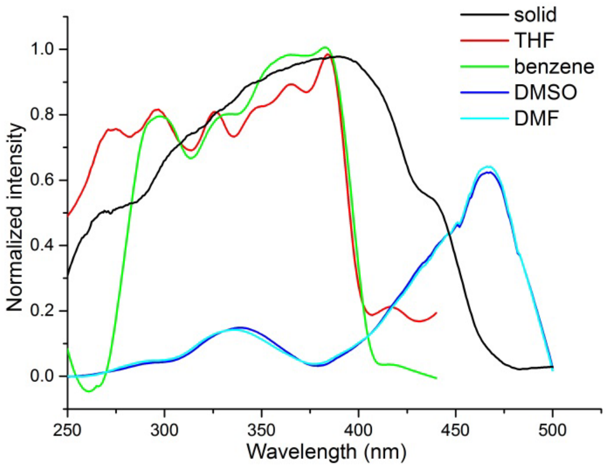

DF2

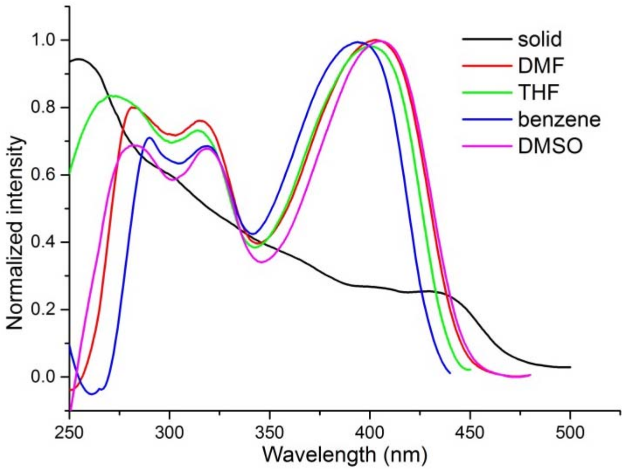

DF3

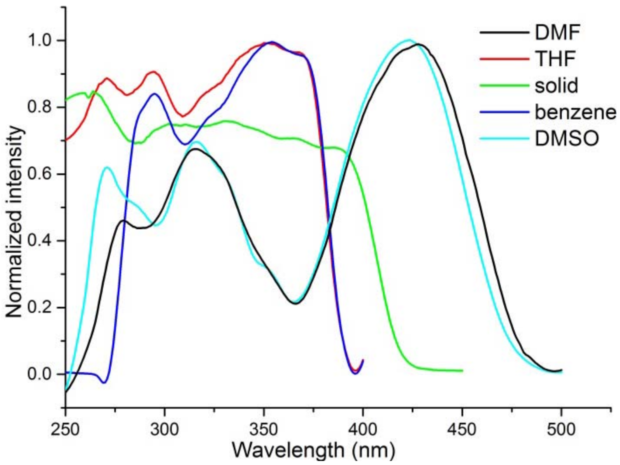

DF4

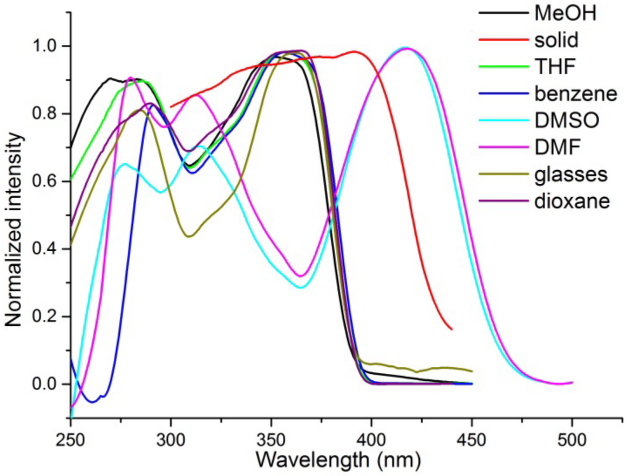

DF5

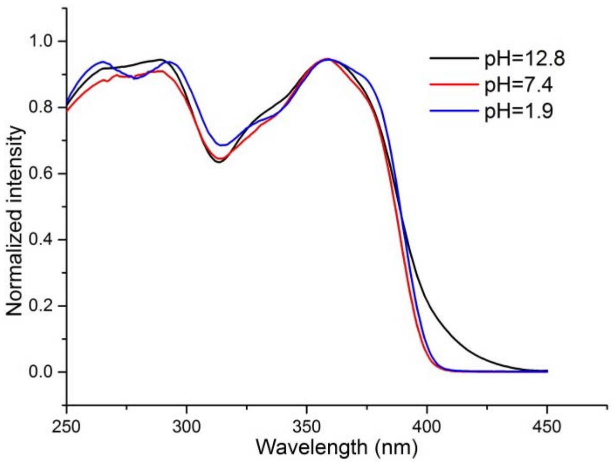

DF5

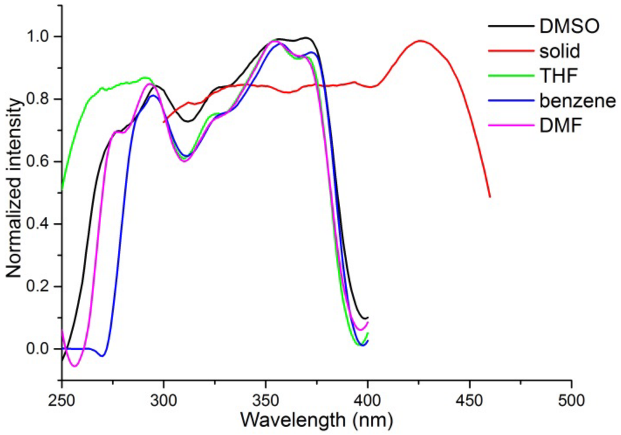

DF6

Figure S9: Normalized excitation spectra of compounds in different solvents (2×10^{-5} M).

8. Emission spectra of the target compounds

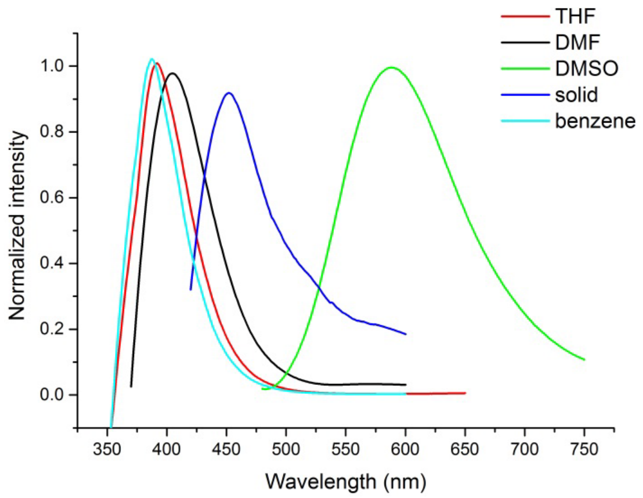

DF0

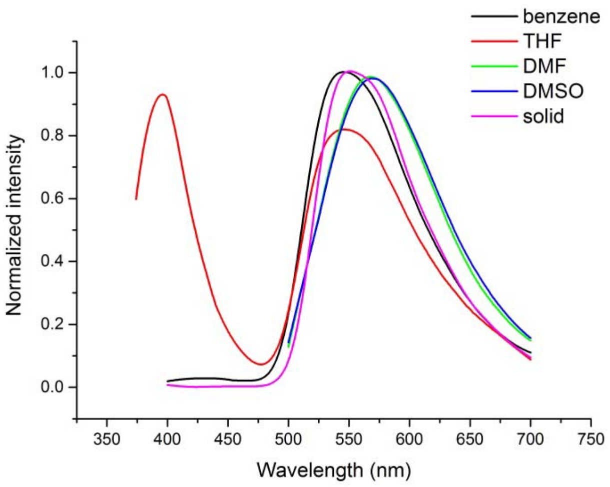

DF1

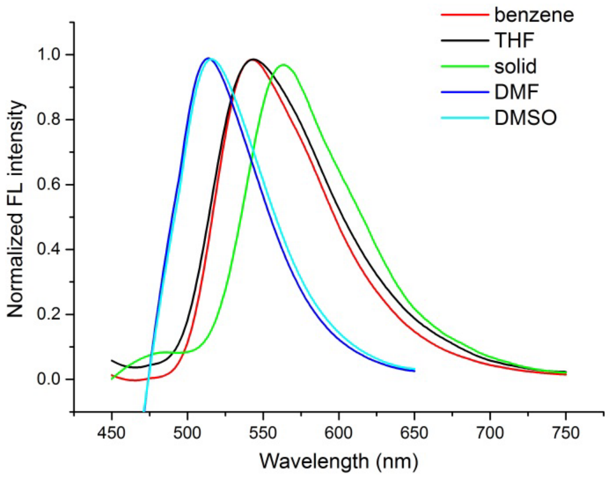

DF2

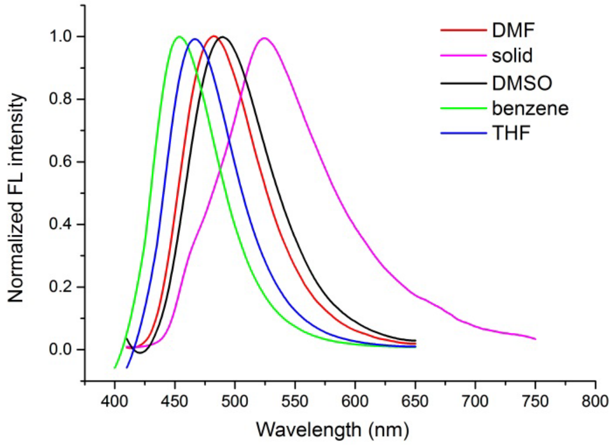

DF3

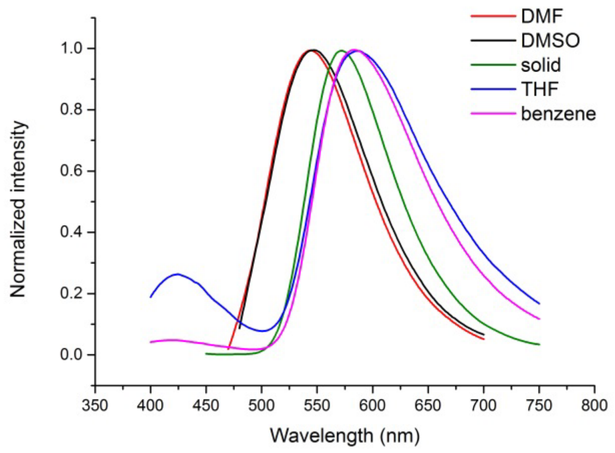

DF4

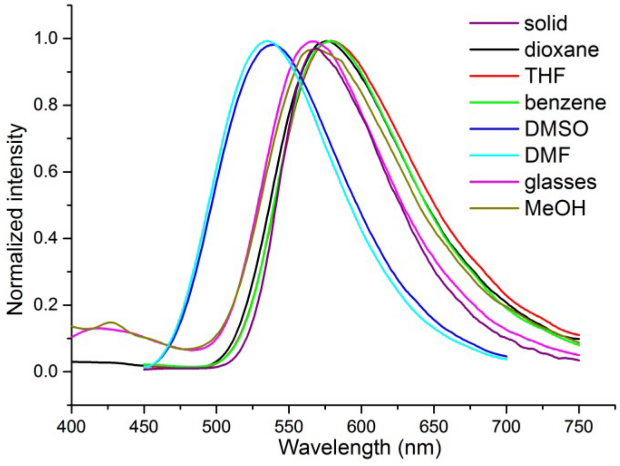

DF5

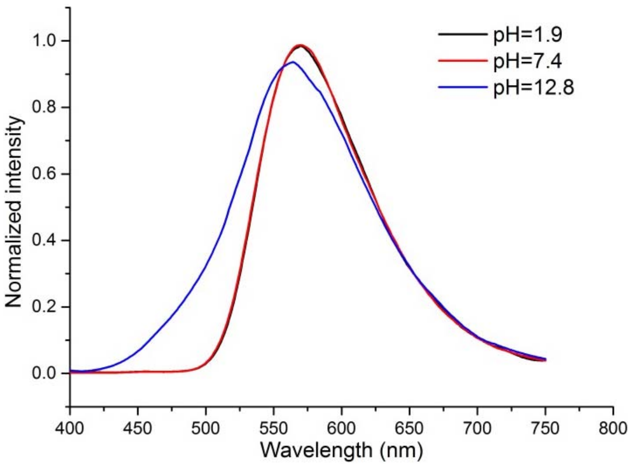

DF5

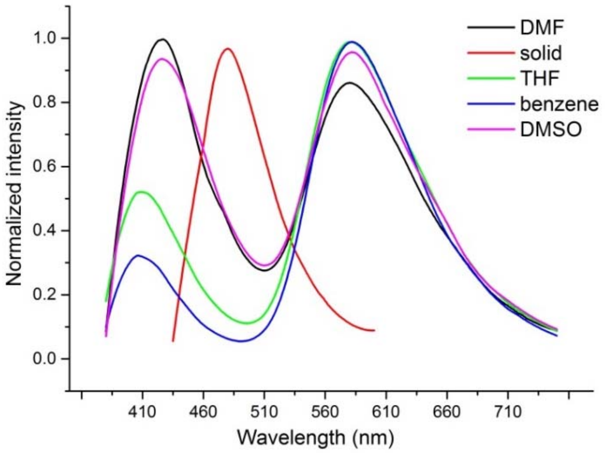

DF6

Figure S10: Normalized emission spectra of compounds in different solvents (2×10^{-5} M).

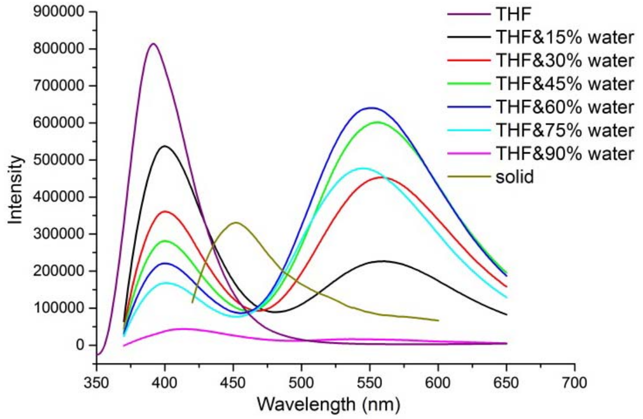

DF0

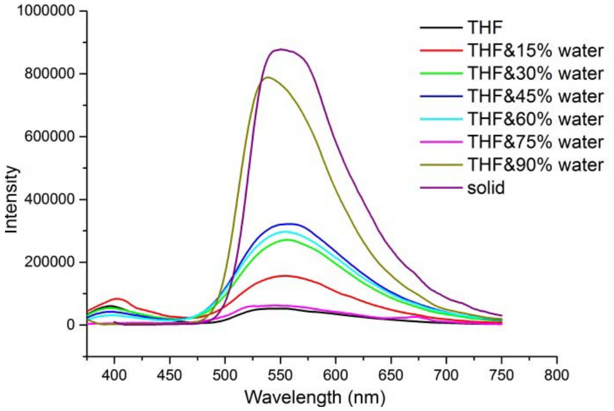

DF1

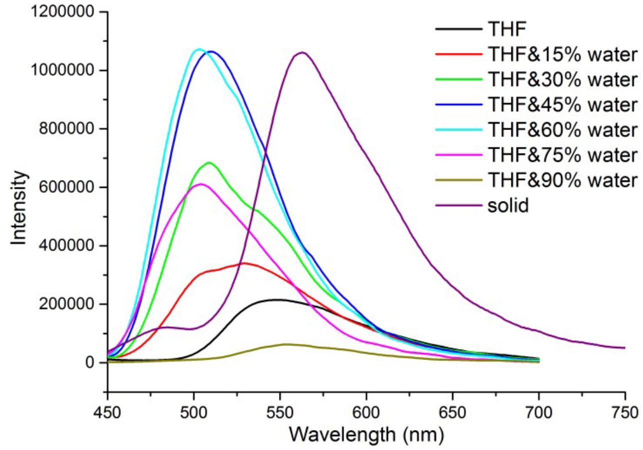

DF2

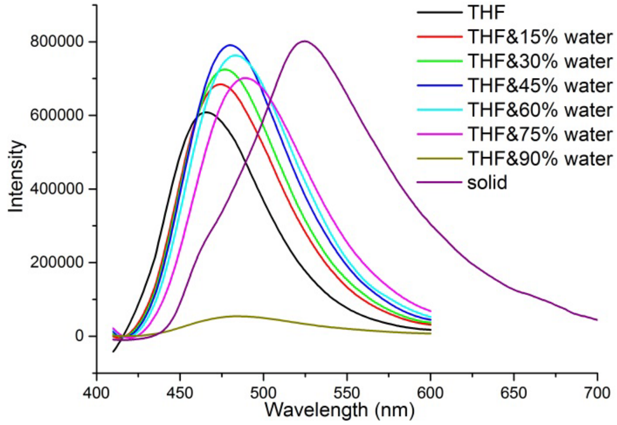

DF3

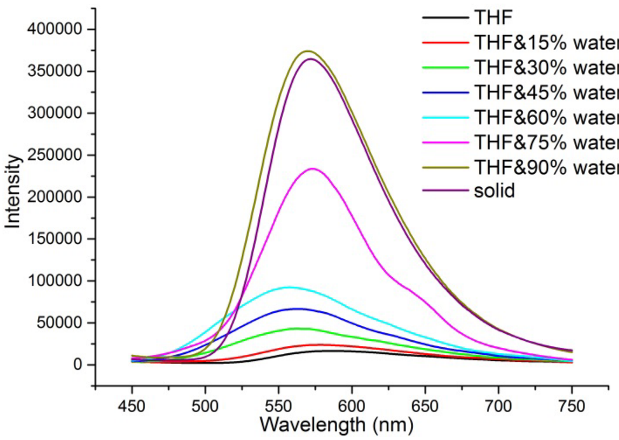

DF4

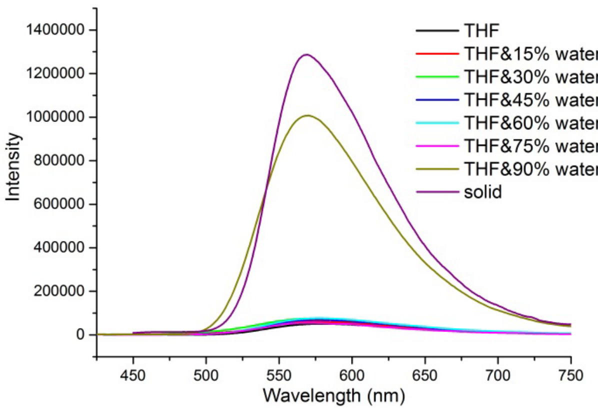

DF5

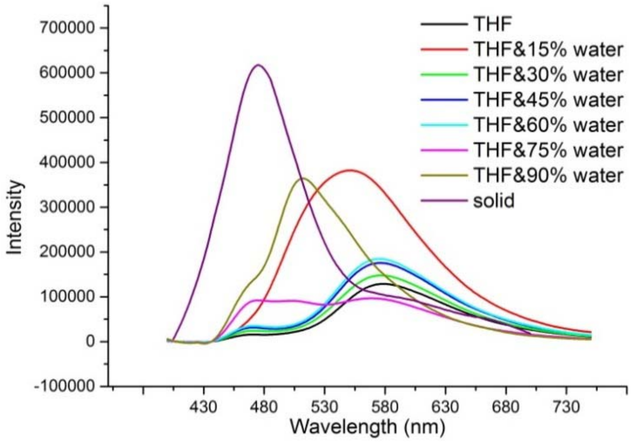

DF6

Figure S11: emission spectra of compounds in THF/water mixtures with different amounts of water (2×10^{-5} M) and solid state.

9. Thermal properties

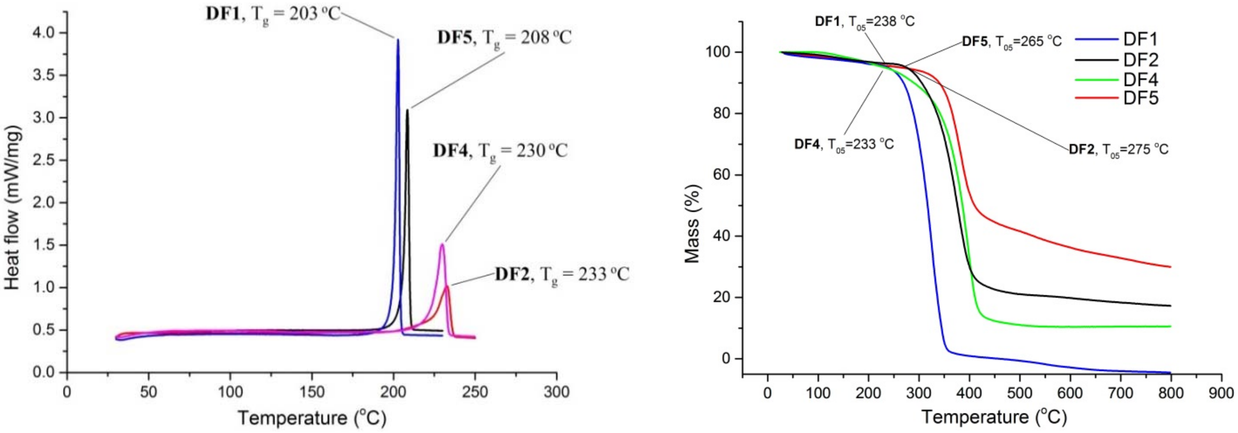

A B

Figure S12: Thermal properties of the compounds. A) DSC B) TGA

Table S2: Thermal properties of the compounds.

Compound	T_{05} / °C	T_m / °C
**DF1**	238	203
**DF2**	275	233
**DF4**	234	230
**DF5**	265	208

10.NMR spectra of the target compounds

S1

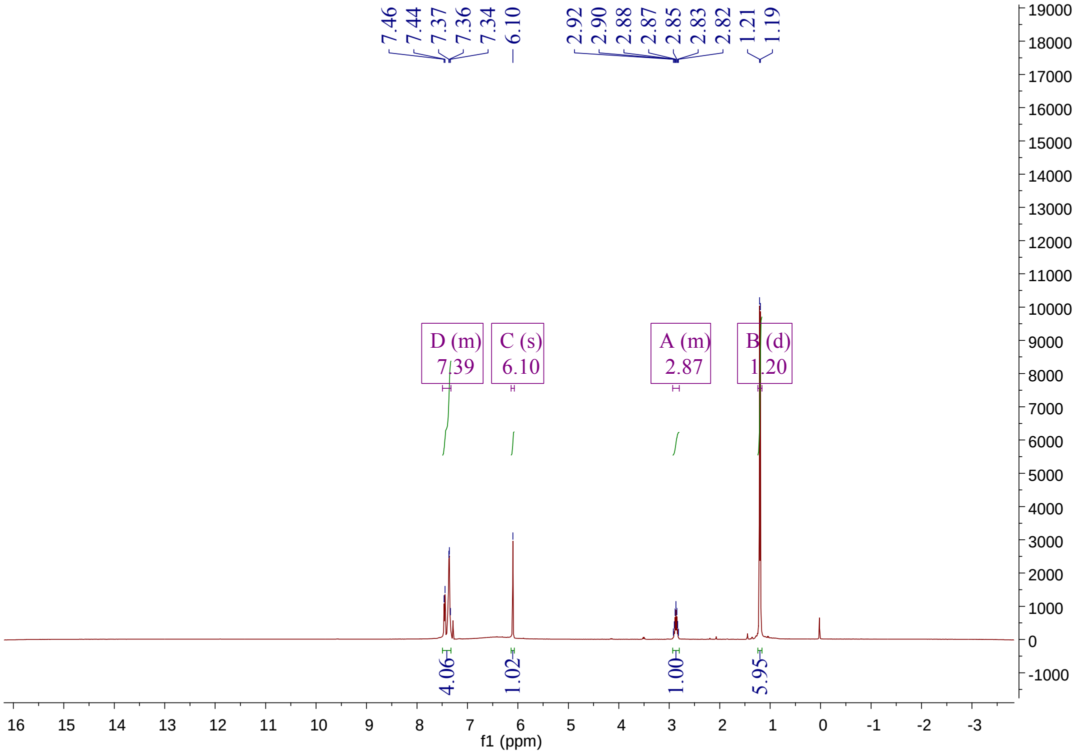

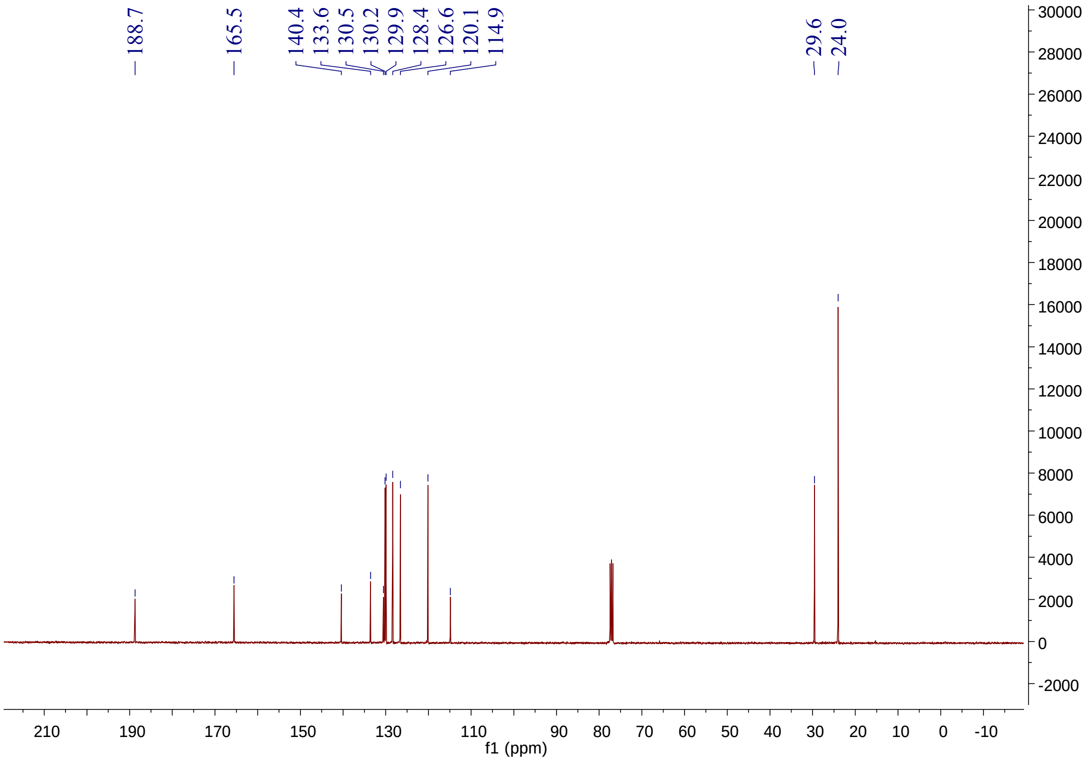

S2

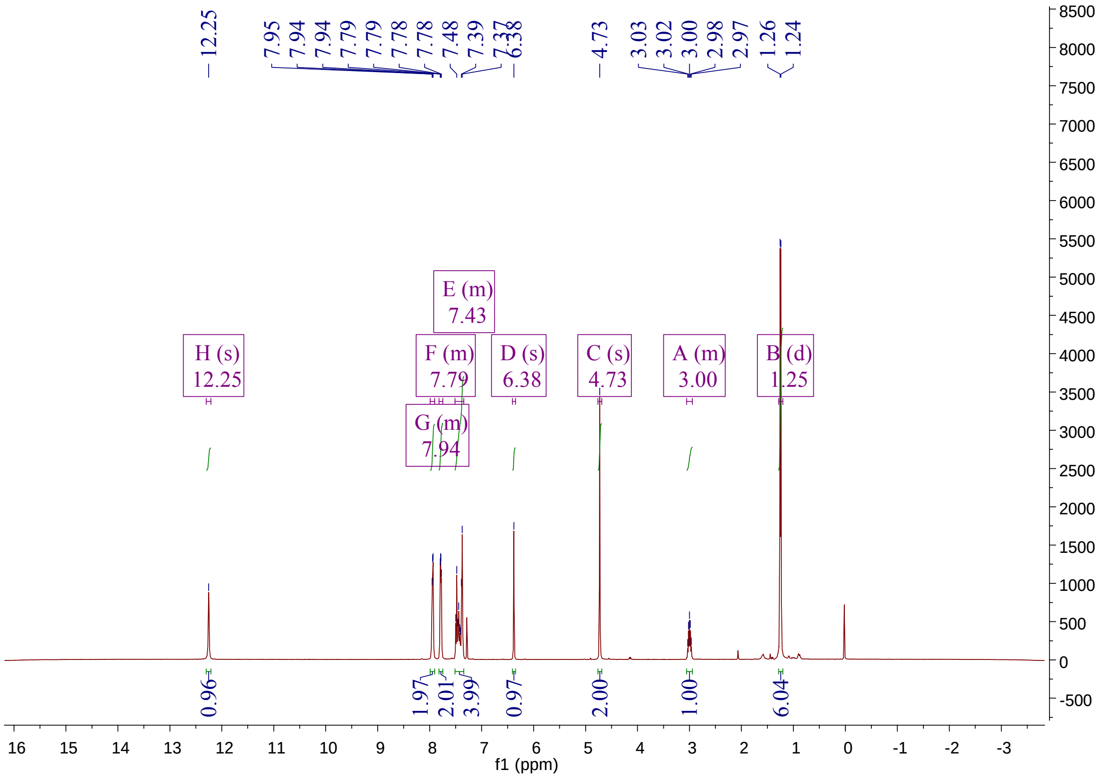
12.25
7.95
7.94
7.94
7.79
7.79
7.78
7.78
7.48
7.39
7.37
6.38
4.73
3.03
3.02
3.00
2.98
2.97
1.26
1.24
E (m)
7.43
H (s)
12.25
F (m)
7.79
D (s)
6.38
C (s)
4.73
A (m)
3.00
B (d)
1.25
G (m)
7.94
0.96
1.97
2.01
3.99
0.97
2.00
1.00
6.04
f1 (ppm)

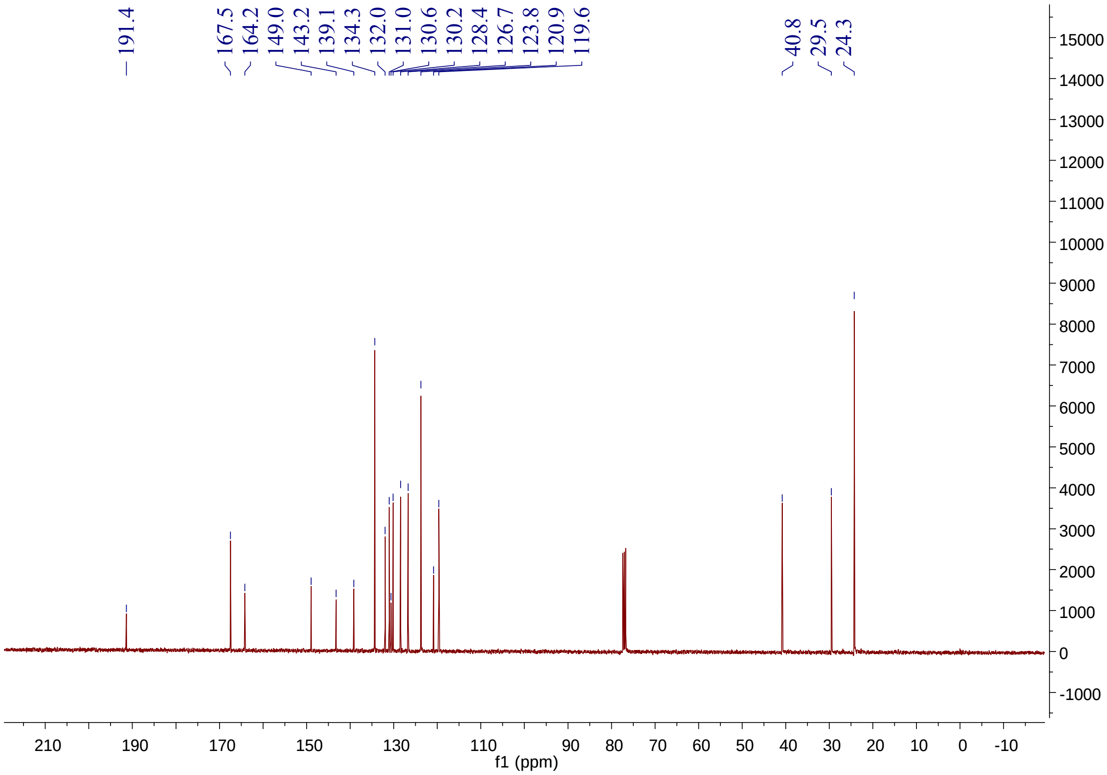
191.4
167.5
164.2
149.0
143.2
139.1
134.3
132.0
131.0
130.6
130.2
128.4
126.7
123.8
120.9
119.6
40.8
29.5
24.3
f1 (ppm)

S3

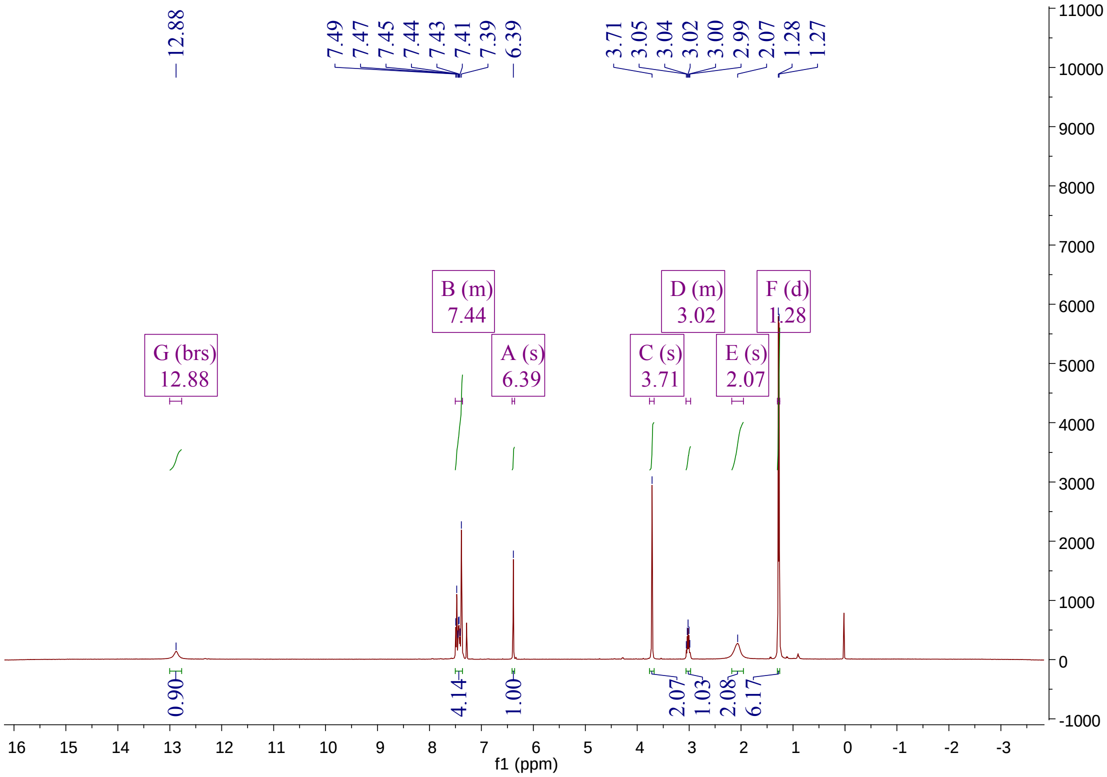
12.88
7.49
7.47
7.45
7.44
7.43
7.41
7.39
6.39
3.71
3.05
3.04
3.02
3.00
2.99
2.07
1.28
1.27
G (brs) 12.88
B (m) 7.44
A (s) 6.39
C (s) 3.71
D (m) 3.02
E (s) 2.07
F (d) 1.28
0.90
4.14
1.00
2.07
1.03
2.08
6.17
f1 (ppm)

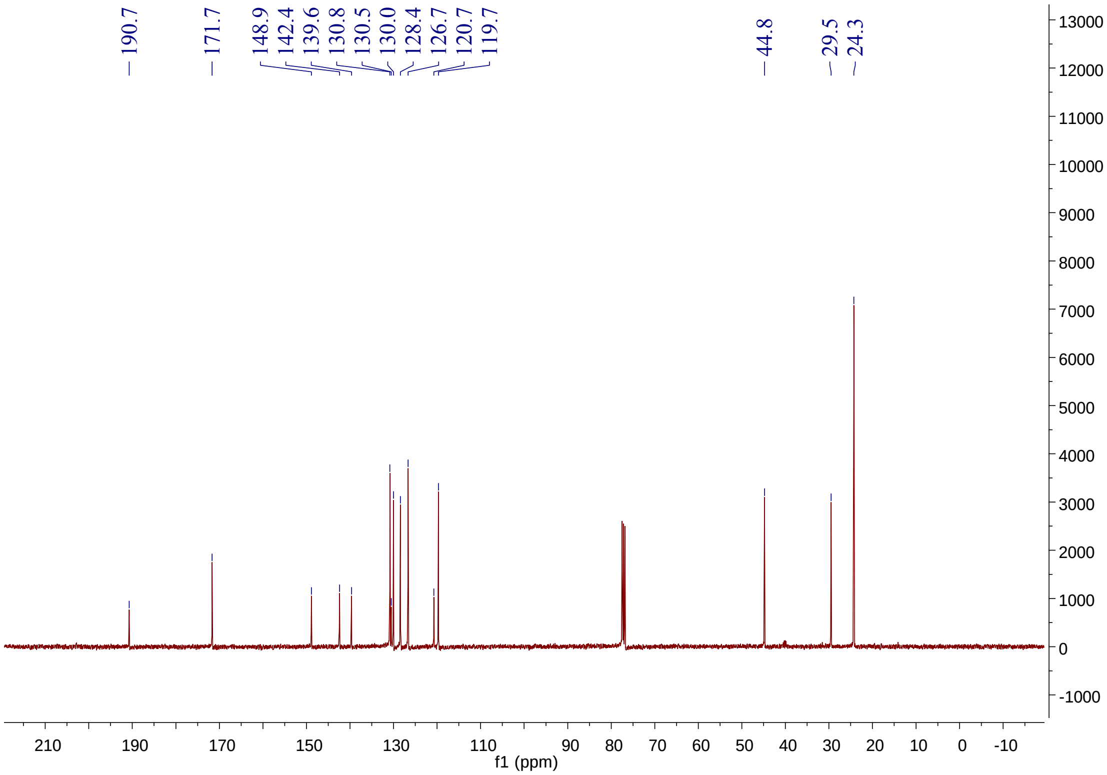
190.7
171.7
148.9
142.4
139.6
130.8
130.5
130.0
128.4
126.7
120.7
119.7
44.8
29.5
24.3
f1 (ppm)

S4

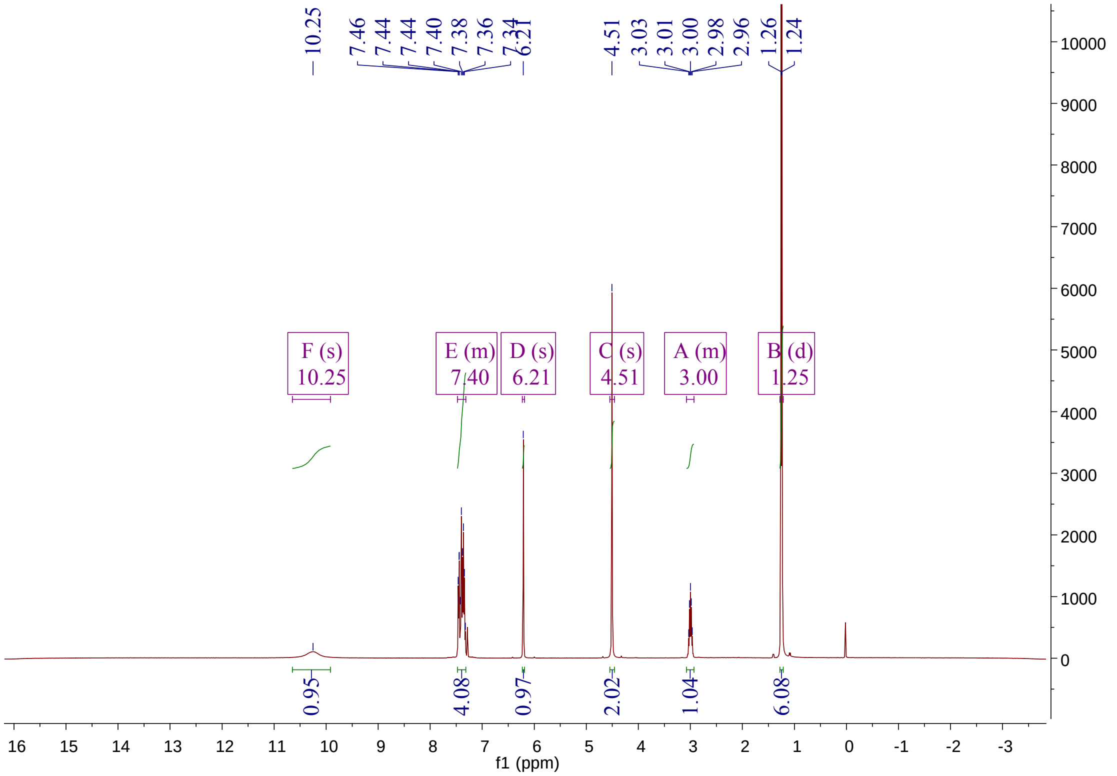
10.25
7.46
7.44
7.44
7.40
7.38
7.36
7.34
6.21
4.51
3.03
3.01
3.00
2.98
2.96
1.26
1.24
F (s) 10.25
E (m) 7.40
D (s) 6.21
C (s) 4.51
A (m) 3.00
B (d) 1.25
0.95
4.08
0.97
2.02
1.04
6.08
f1 (ppm)

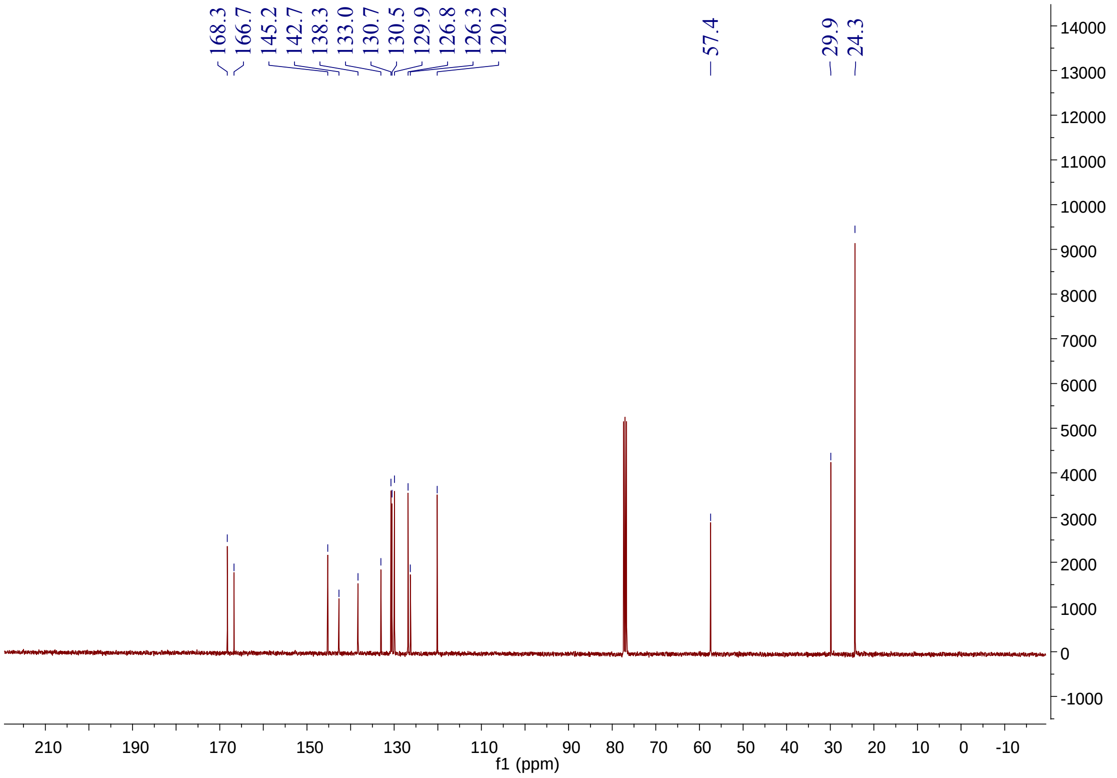
168.3
166.7
145.2
142.7
138.3
133.0
130.7
130.5
129.9
126.8
126.3
120.2
57.4
29.9
24.3
f1 (ppm)

DF

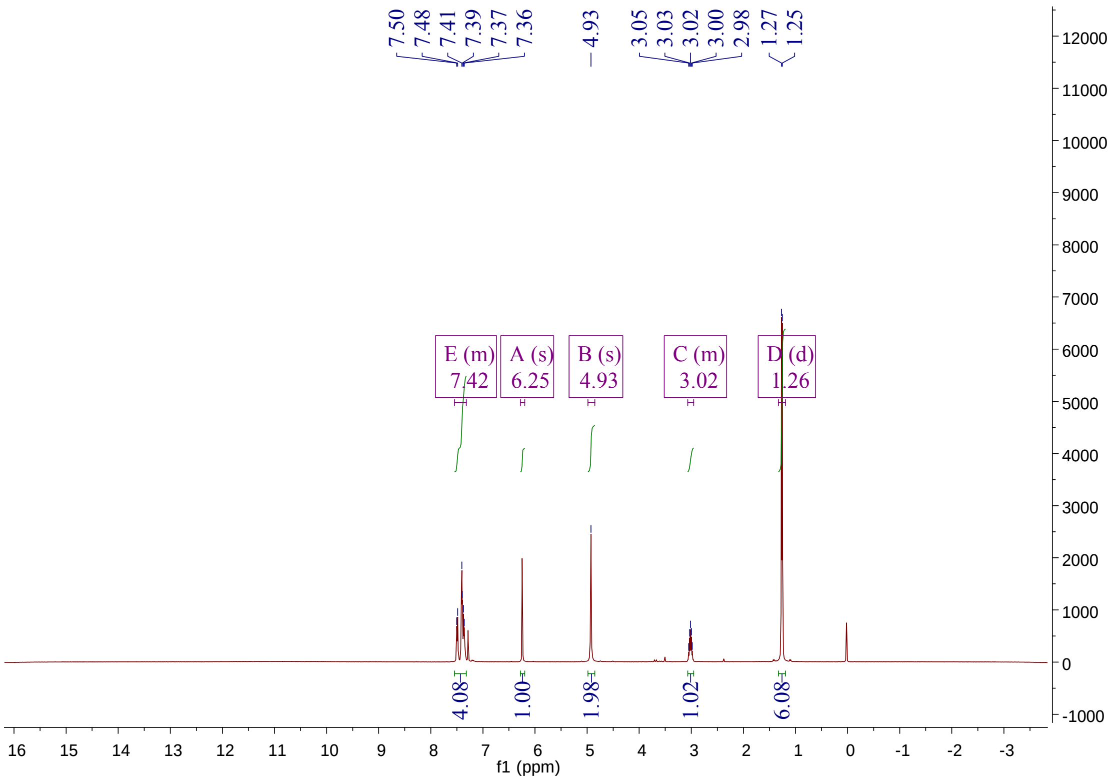
7.50
7.48
7.41
7.39
7.37
7.36
4.93
3.05
3.03
3.02
3.00
2.98
1.27
1.25
E (m) 7.42
A (s) 6.25
B (s) 4.93
C (m) 3.02
D (d) 1.26
4.08
1.00
1.98
1.02
6.08
f1 (ppm)

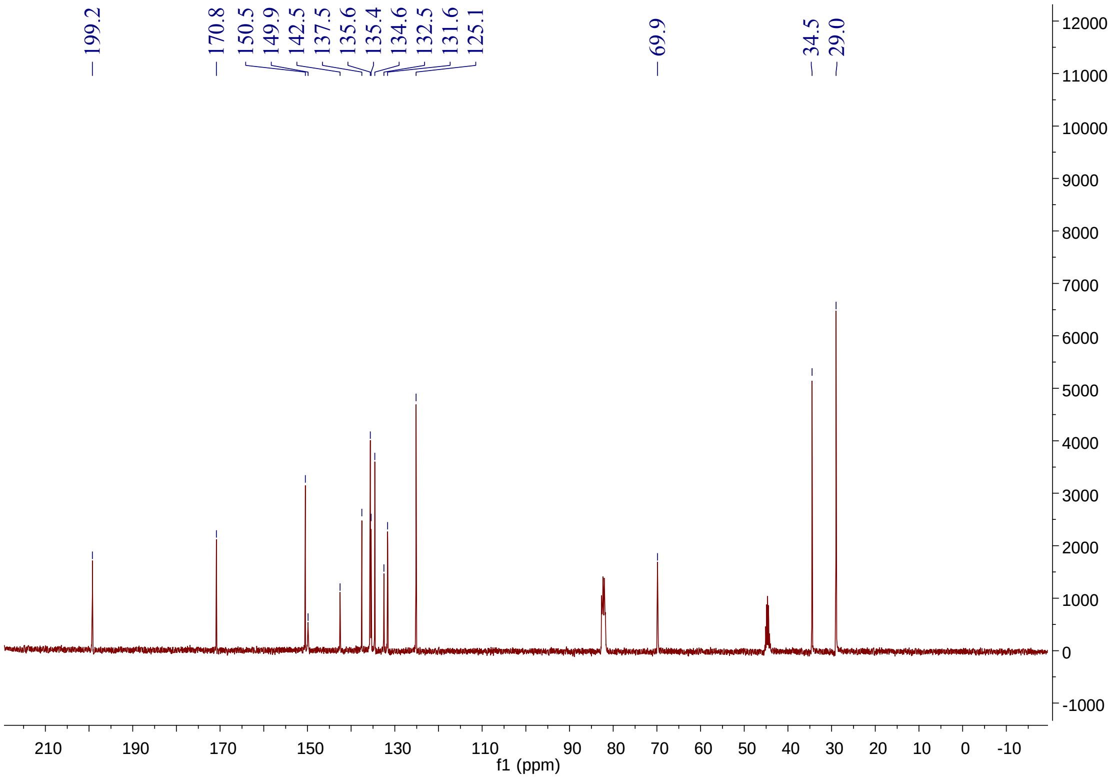
199.2
170.8
150.5
149.9
142.5
137.5
135.6
135.4
134.6
132.5
131.6
125.1
69.9
34.5
29.0
f1 (ppm)

DF1

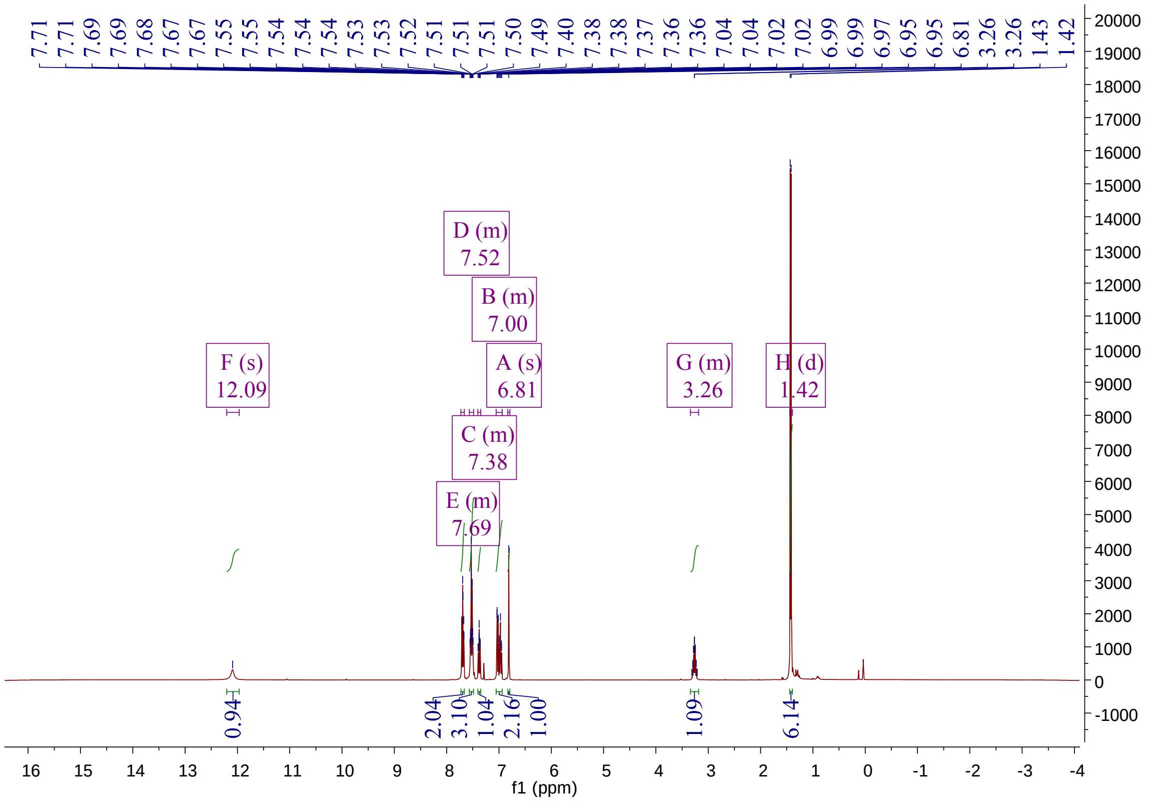
7.71 7.71 7.69 7.69 7.68 7.67 7.67 7.55 7.55 7.54 7.54 7.54 7.53 7.53 7.52 7.51 7.51 7.51 7.50 7.49 7.40 7.38 7.38 7.37 7.36 7.36 7.04 7.04 7.02 7.02 6.99 6.99 6.97 6.95 6.95 6.81 3.26 3.26 1.43 1.42
D (m) 7.52
B (m) 7.00
F (s) 12.09
A (s) 6.81
G (m) 3.26
H (d) 1.42
C (m) 7.38
E (m) 7.69
0.94 2.04 3.10 1.04 2.16 1.00 1.09 6.14
f1 (ppm)

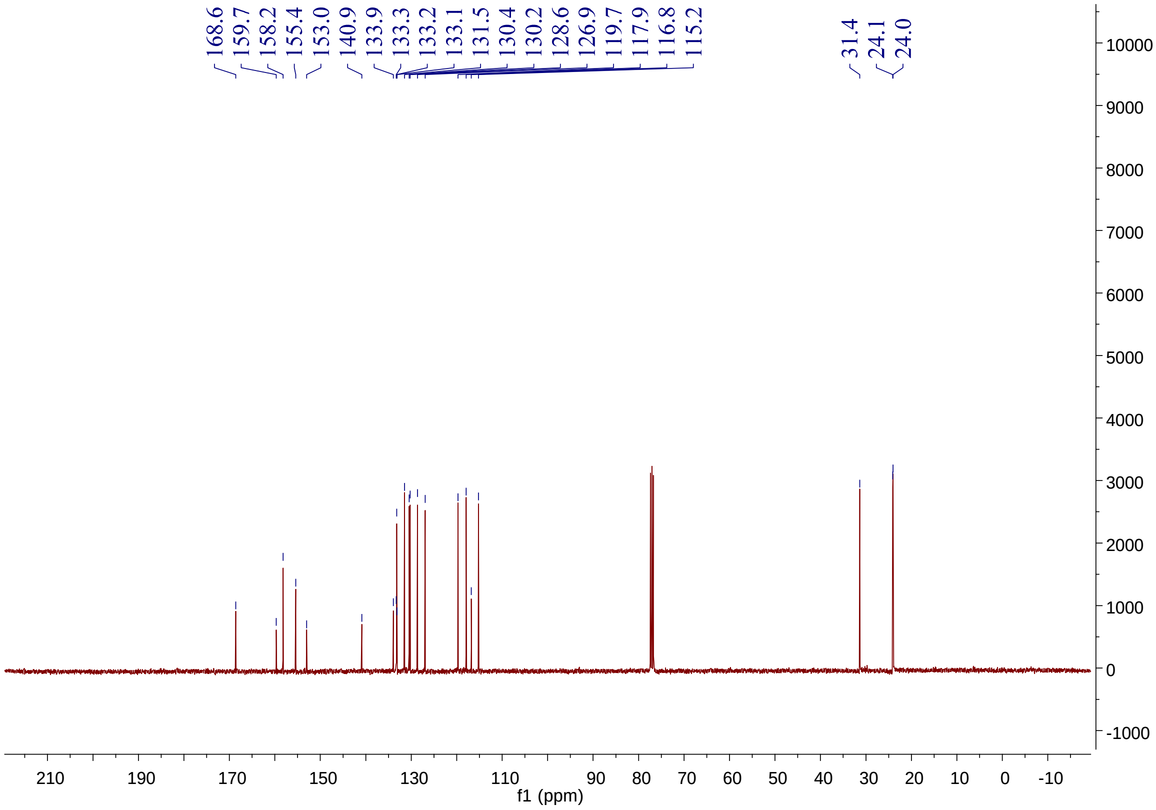
168.6 159.7 158.2 155.4 153.0 140.9 133.9 133.3 133.2 133.1 131.5 130.4 130.2 128.6 126.9 119.7 117.9 116.8 115.2
31.4 24.1 24.0
f1 (ppm)

DF2

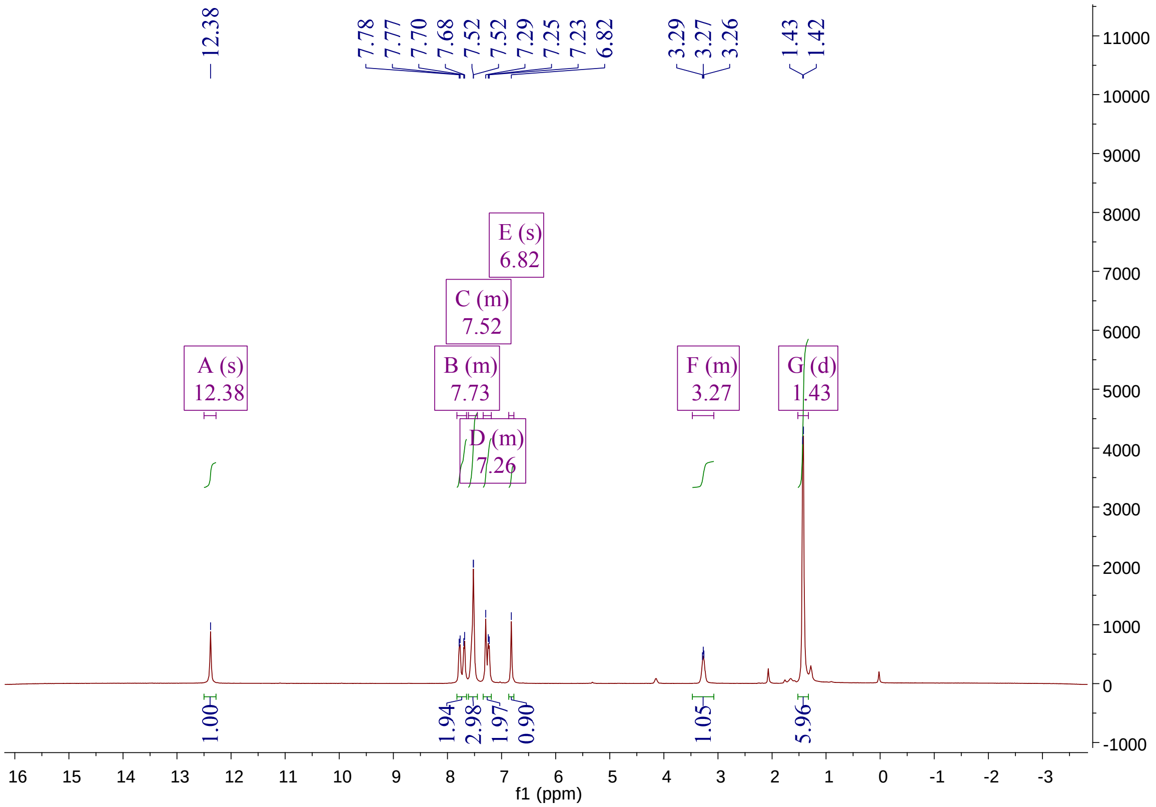
12.38
7.78 7.77 7.70 7.68 7.52 7.52 7.29 7.25 7.23 6.82
3.29 3.27 3.26
1.43 1.42
A (s) 12.38
B (m) 7.73
C (m) 7.52
D (m) 7.26
E (s) 6.82
F (m) 3.27
G (d) 1.43
1.00
1.94 2.98 1.97 0.90
1.05
5.96
f1 (ppm)

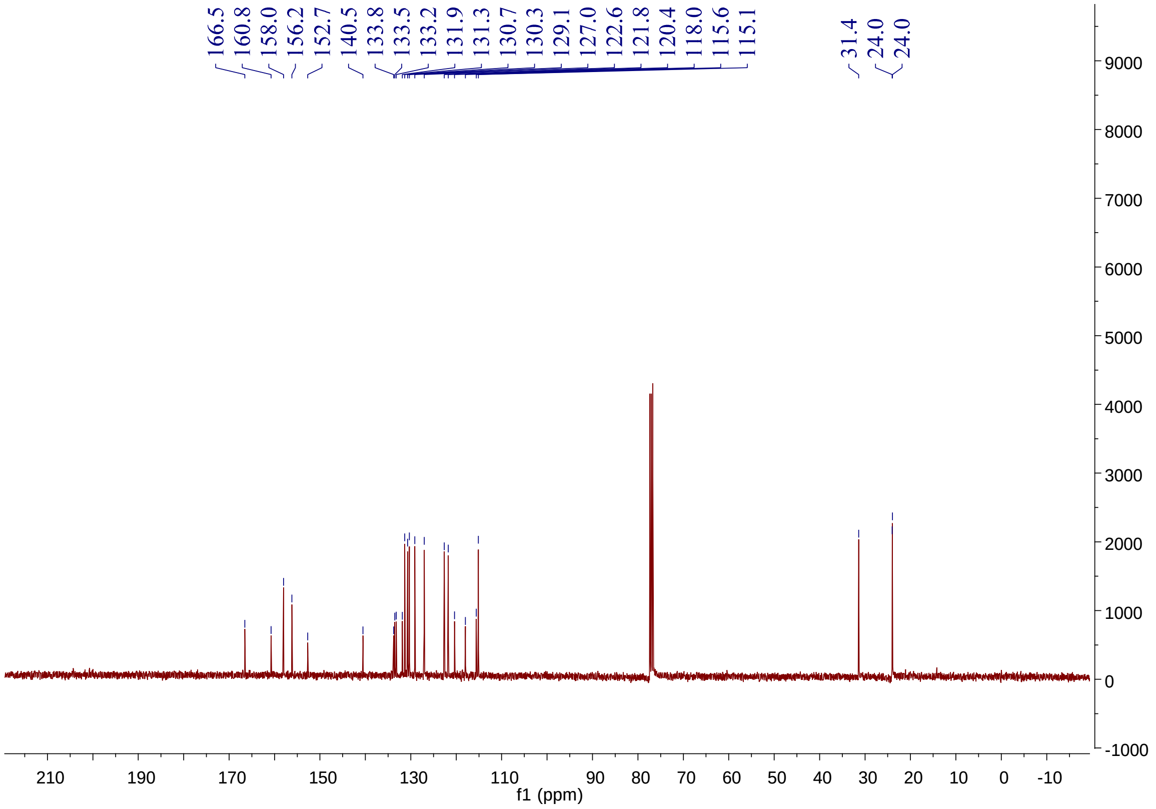
166.5 160.8 158.0 156.2 152.7 140.5 133.8 133.5 133.2 131.9 131.3 130.7 130.3 129.1 127.0 122.6 121.8 120.4 118.0 115.6 115.1
31.4 24.0 24.0
f1 (ppm)

DF3

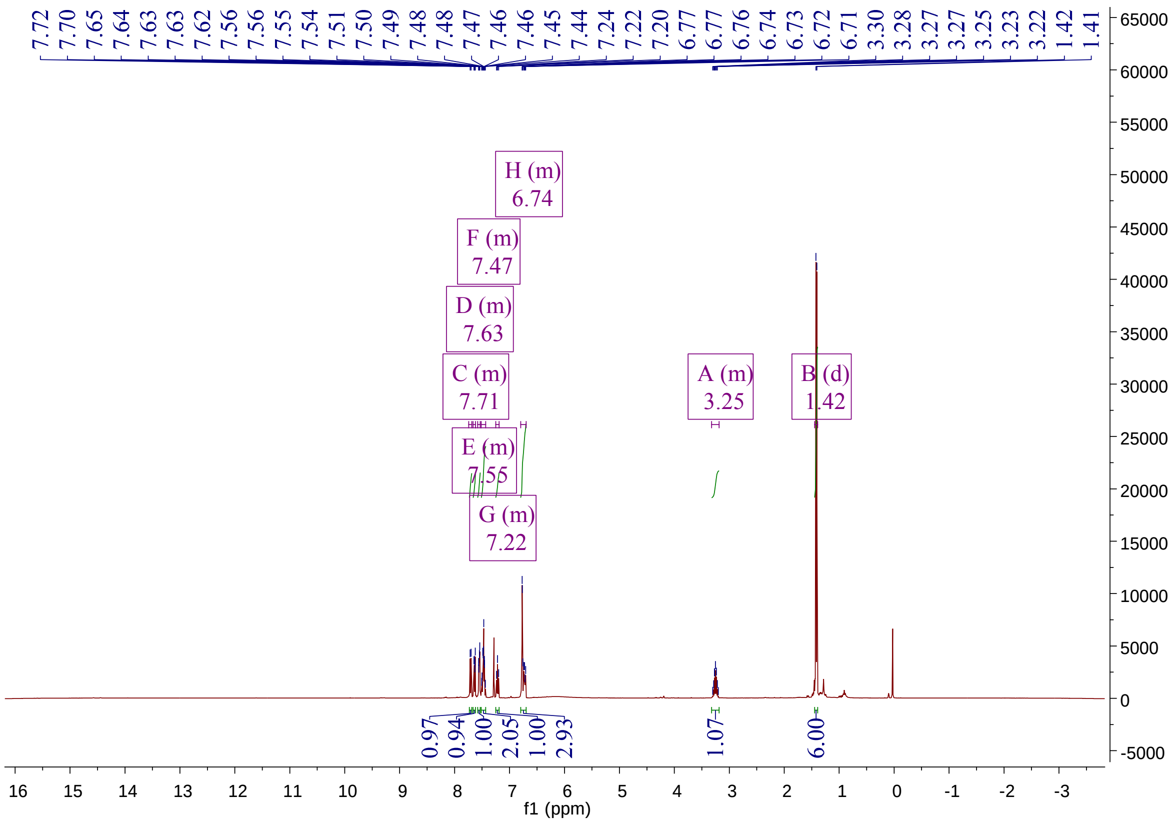

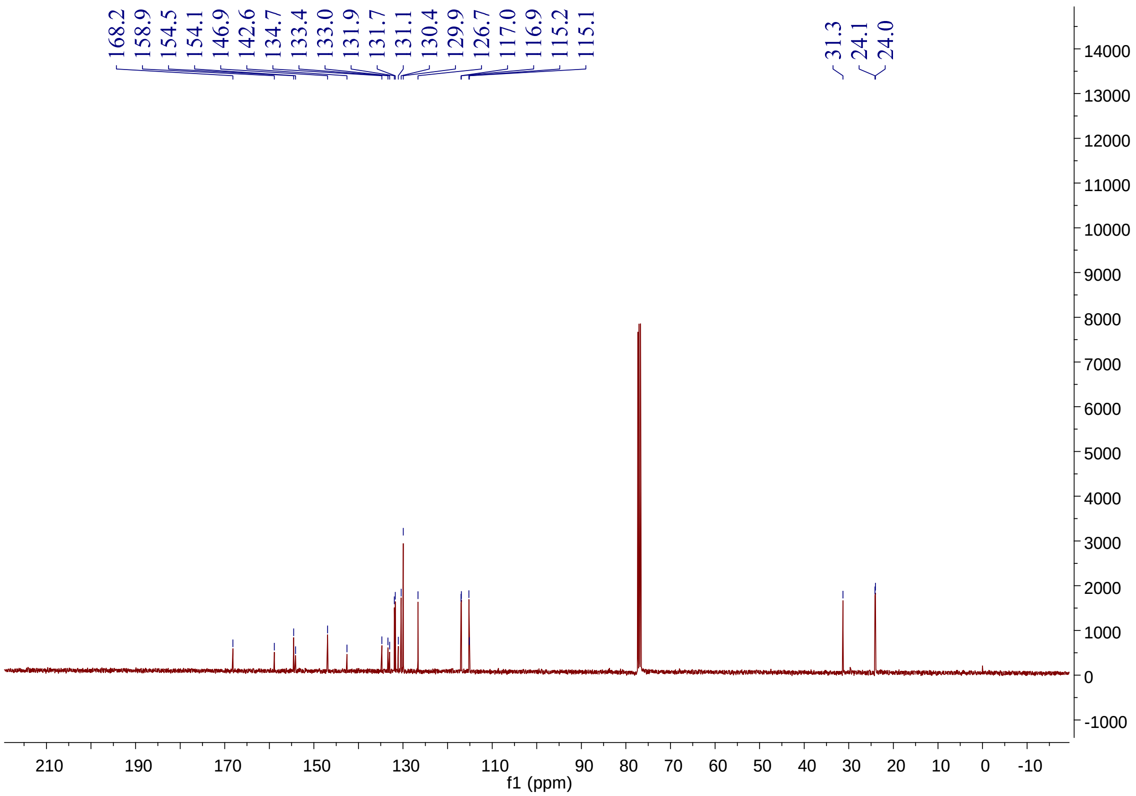

DF4

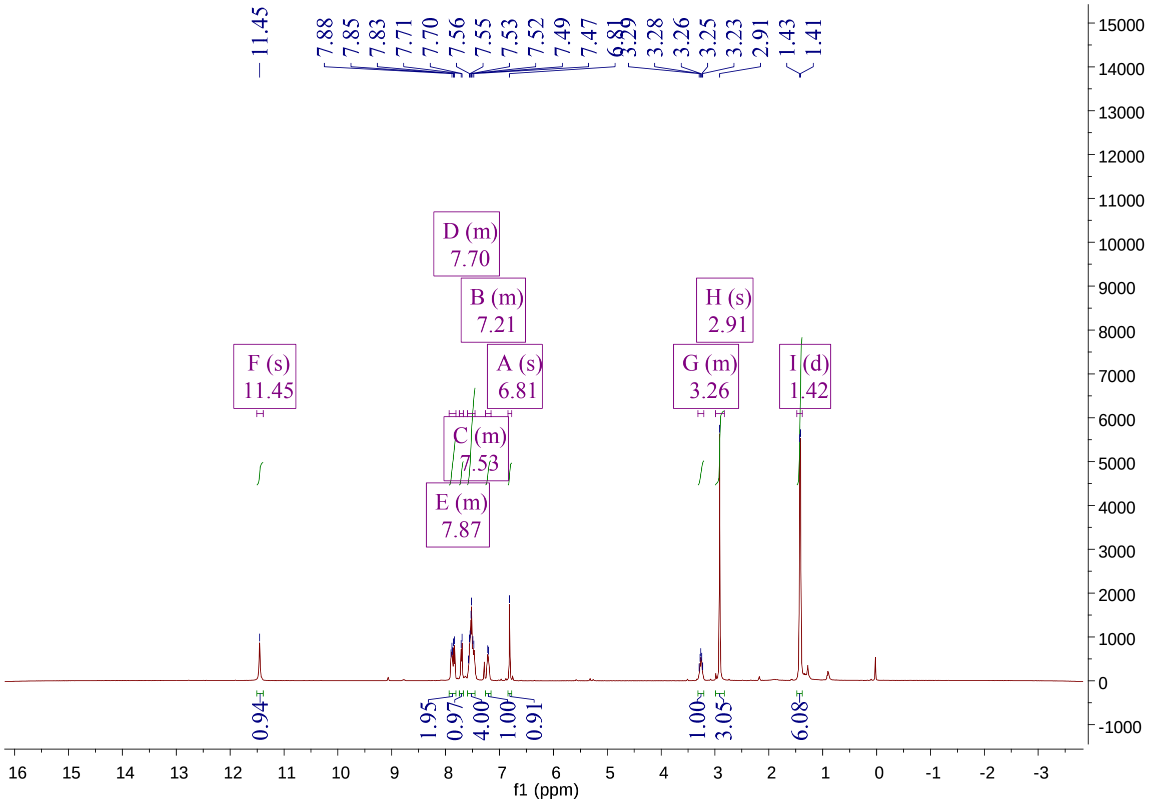
11.45
7.88
7.85
7.83
7.71
7.70
7.56
7.55
7.53
7.52
7.49
7.47
6.81
3.29
3.28
3.26
3.25
3.23
2.91
1.43
1.41
D (m) 7.70
B (m) 7.21
H (s) 2.91
F (s) 11.45
A (s) 6.81
G (m) 3.26
I (d) 1.42
C (m) 7.53
E (m) 7.87
0.94
1.95
0.97
4.00
1.00
0.91
1.00
3.05
6.08
f1 (ppm)

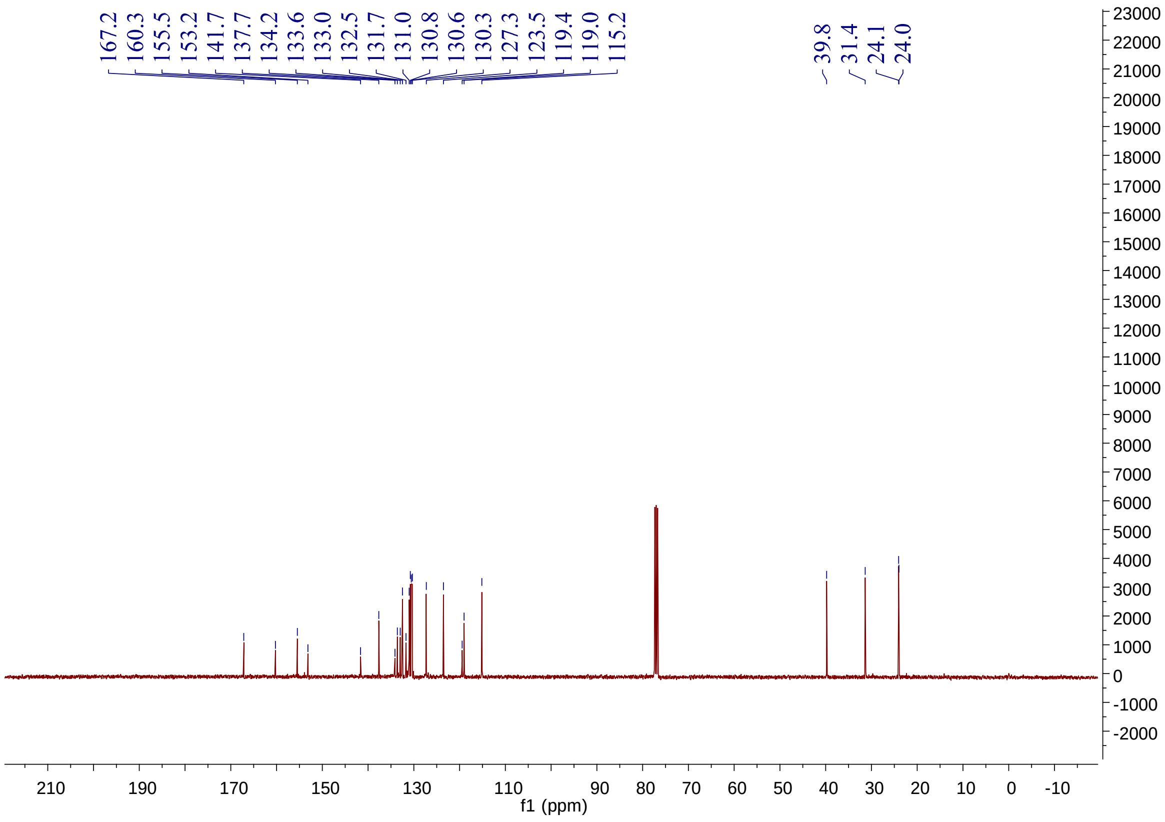
167.2
160.3
155.5
153.2
141.7
137.7
134.2
133.6
133.0
132.5
131.7
131.0
130.8
130.6
130.3
127.3
123.5
119.4
119.0
115.2
39.8
31.4
24.1
24.0
f1 (ppm)

DF5

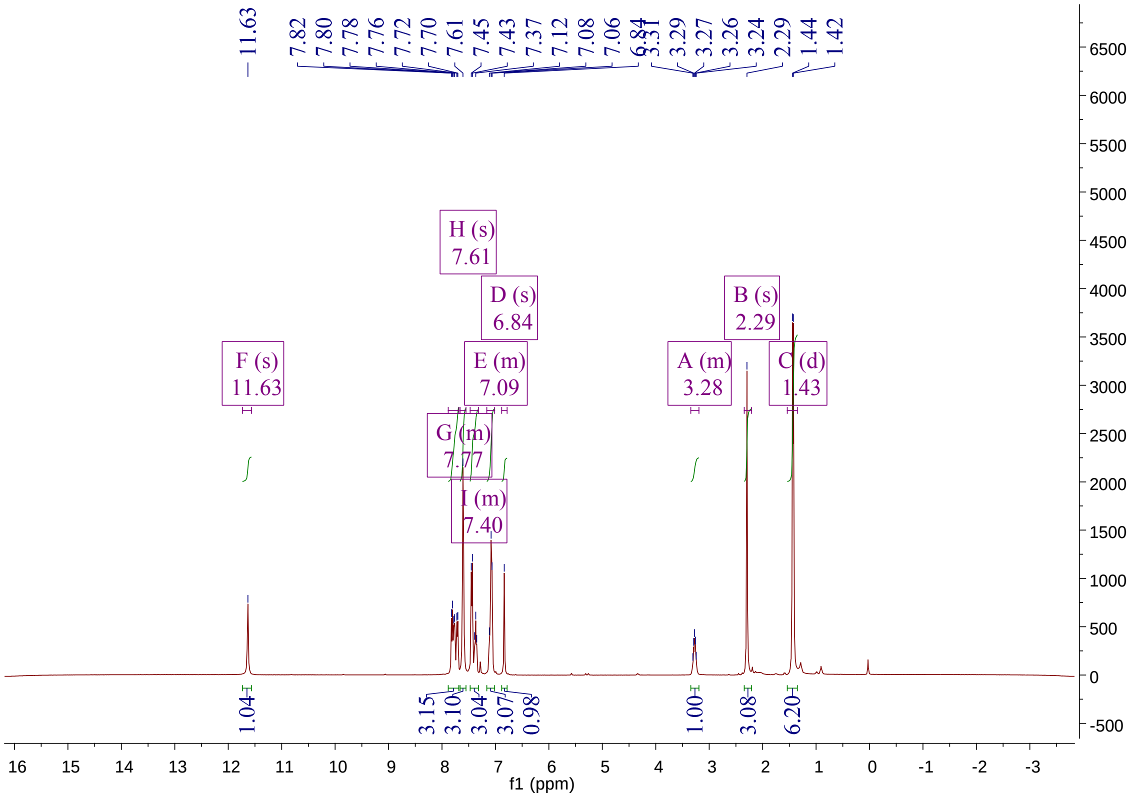
11.63
7.82
7.80
7.78
7.76
7.72
7.70
7.61
7.45
7.43
7.37
7.12
7.08
7.06
6.84
3.31
3.29
3.27
3.26
3.24
2.29
1.44
1.42
H (s)
7.61
D (s)
6.84
B (s)
2.29
F (s)
11.63
E (m)
7.09
A (m)
3.28
C (d)
1.43
G (m)
7.77
I (m)
7.40
1.04
3.15
3.10
3.04
3.07
0.98
1.00
3.08
6.20
f1 (ppm)

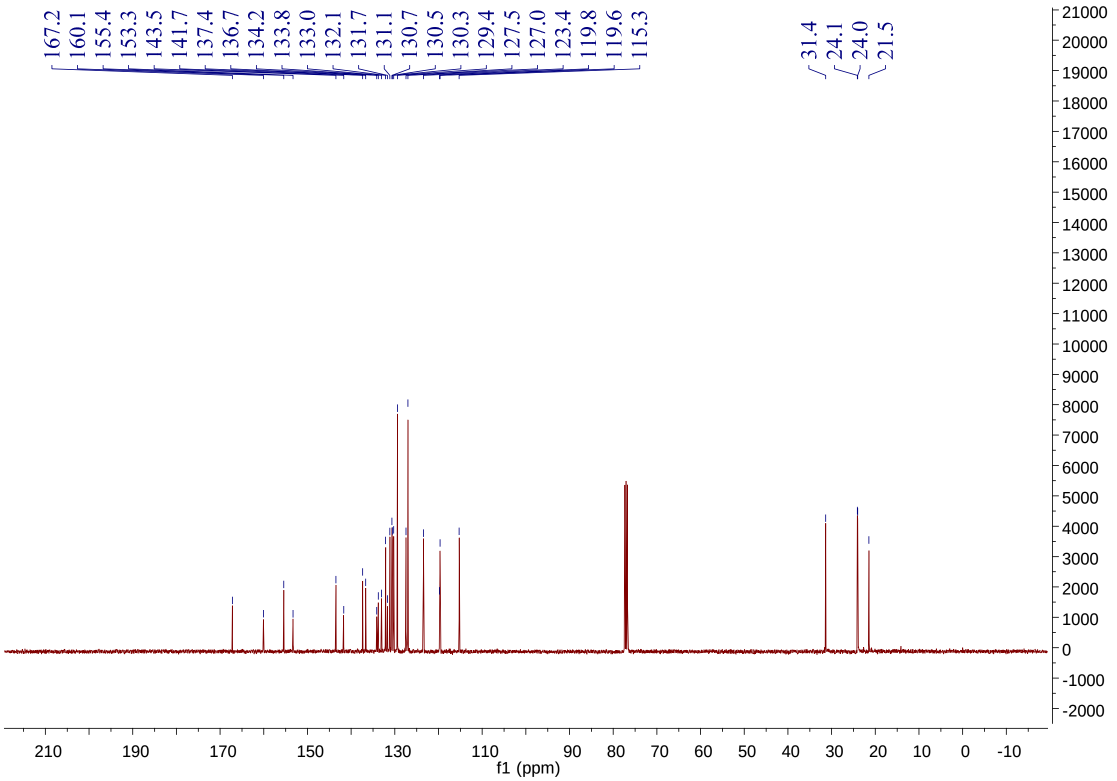
167.2
160.1
155.4
153.3
143.5
141.7
137.4
136.7
134.2
133.8
133.0
132.1
131.7
131.1
130.7
130.5
130.3
129.4
127.5
127.0
123.4
119.8
119.6
115.3
31.4
24.1
24.0
21.5
f1 (ppm)

DF6

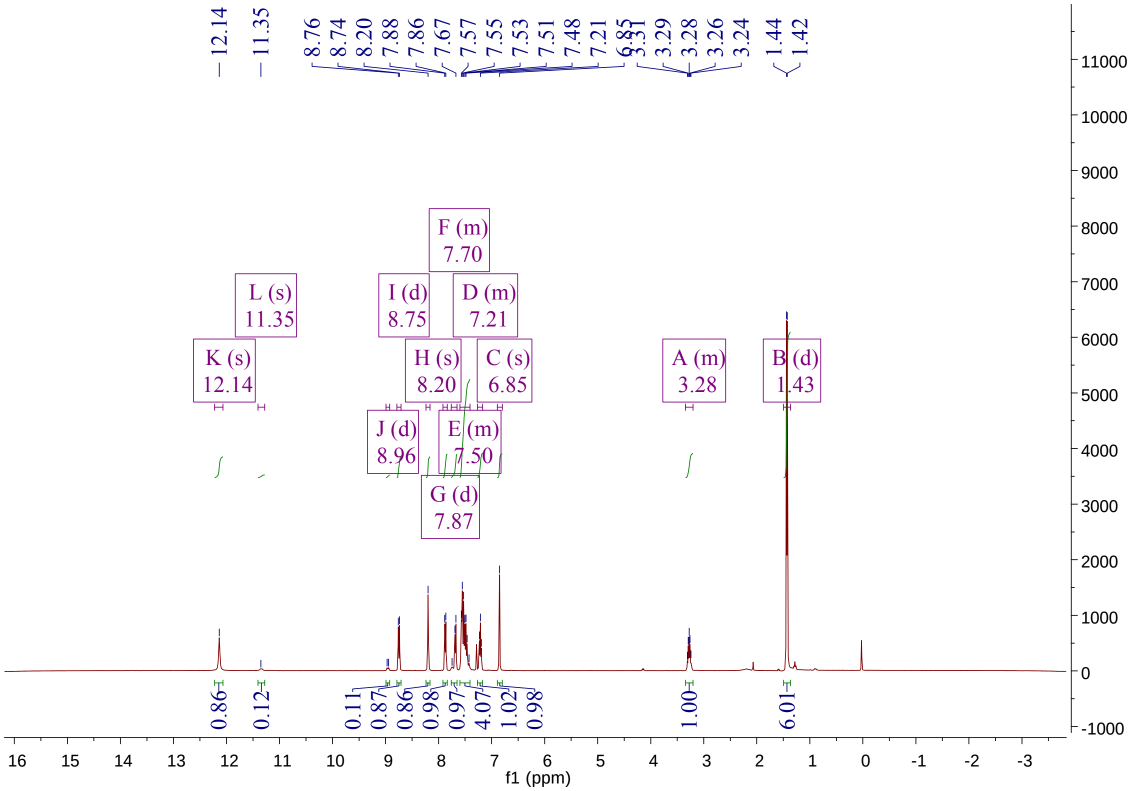

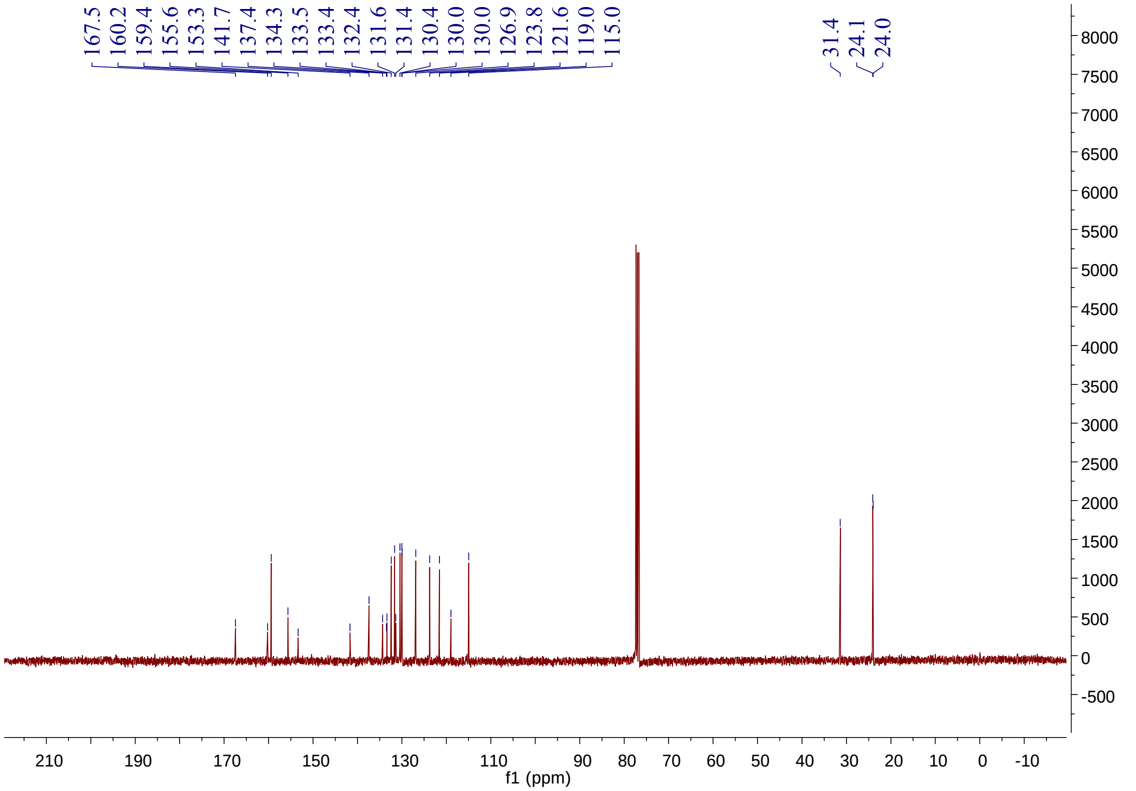

11. References

[1] L. Huang, R. Yu, L. Leng, F. Gong, X. Zhu, Y. Wan, *Synthesis* **2014**, *46*, 2317-2326.

[2] H. J. Tracy, J. L. Mullin, W. T. Klooster, J. A. Martin, J. Haug, S. Wallace, I. Rudloe, K. Watts, *Inorg. Chem.* **2005**, *44*, 2003-2011.
